# Supplementary material for: Escherichia coli tRNA 2-Selenouridine Synthase (SelU): Elucidation of Substrate Specificity to Understand the Role of S-Geranyl-tRNA in the Conversion of 2-Thio- into 2-Selenouridines in Bacterial tRNA
Source: Cells. 2022 May 2;11(9):1522. doi: 10.3390/cells11091522 (PMC9105526; doi:10.3390/cells11091522)
Supplement: Supplementary file 1 [file cells-11-01522-s001.zip › cells-1698167-supplementary.pdf]

Escherichia coli tRNA 2-selenouridine synthase (SelU); elucidation of its substrate specificity for understanding the role of _S_-geranyl-tRNA in the conversion of 2-thio- to 2-selenouridines in bacterial tRNA

Patrycja Szczupak[1], Malgorzata Sierant*[1], Ewelina Wielgus[1], Ewa Radzikowska-Cieciura[1], Katarzyna Kulik[1], Agnieszka Krakowiak[1], Paulina Kuwerska[2], Grazyna Leszczynska[2], Barbara Nawrot[1]

1. Centre of Molecular and Macromolecular Studies, Polish Academy of Sciences, Lodz 90-363, Sienkiewicza 112, Poland
2. Institute of Organic Chemistry, Lodz University of Technology, Lodz 90-924, Zeromskiego 116, Poland

* Correspondence: msierant@cbmm.lodz.pl; Tel: +48 (42) 680-32-72

SUPPLEMENTARY INFORMATION

Synthetic procedures

1. Synthesis of a R5-substituted 2-oxo-, 2-thio-, S-geranyl-2-thio- and 2-seleno- uridines.

2. Synthesis of S2U and geS2U phosphoroamidites:

3. Synthesis of model RNA oligonucleotides.

4. Synthesis of selenophosphate $SePO_3^{3-}$.

Protein procedures:

Synthesis and purification of MBP-SelU,

Tables

Table S1 Sequences and ESI-MS characteristic of the model ASL-RNAs used in studies.

Table S2 Detailed conditions for S2U-RNA geranylation and geS2U-RNA selenation reactions catalyzed by MBP-SelU.

Table S3 Summary of kinetic data of geranylation and selenation reactions catalyzed by MBP-SelU.

Table S4 MST Dataset Overview.

Table S5 Calculation of the molecular formula and molecular weight of the bacterial $tRNA^{Lys}$, $tRNA^{Glu}$ and $tRNA^{Gln}$ depending on nucleoside modification at the wobble position.

Table S6 Qualitative analysis of modified nucleosides present in the wobble position of tRNAs associated with MBP-SelU protein, results of UPLC-PDA-ESI(-)-HRMS measurements.

Table S7 Results of UPLC-PDA-ESI(-)-HRMS measurements, identification of additional (present in a position other than the wobble) modified nucleosides in the bacterial tRNAs specific for Lys, Glu and Gln associated with the MBP-SelU protein.

Schemes

Scheme S1 Synthetic pathway of 5-aminomethyl-_S_-geranyl-2-thiouridine (nm5geS2U).

Scheme S2 Synthetic pathway of 5-aminomethyl-2-selenouridine (nm5Se2U).

Figures

Figure S1 Overexpression and purification of MBP-SelU protein.

Figure S2 The separation of the pure proteins from the mixture of MBP-SelU and free MBP by gel filtration on the Superdex 200 resin.

Figure S3 ESI-TOF MS analysis of the oligo-RNA standards used in the studies.

Figure S4 Kinetic data determined for the reactions catalyzed by MBP-SelU.

Figure S5 The chromatographic mobility of the oligo-RNA standards (3-17-mers).

Figure S6 Geranylation of S2U-RNA substrates with the MBP-SelU enzyme.

Figure S7 Selenation of geS2U-RNA substrates with the MBP-SelU enzyme.

Figure S8 MST Capillary Scan test, the fluorescence intensity changes in the measured sample.

Figure S9 Determination of tRNA bound to MBP-SelU.

Figure S10 Sequences and the positions of the nucleoside modifications in the bacterial tRNAs specific for Lys, Glu and Gln, the underlined sequence corresponds to the anticodon sequence.

Figure S11 The list of nucleoside standards used in studies, their molecular formula and UV characteristics.

Figure S12 Elemental Composition Report for mnm5S2U, mnm5geS2U and mnm5Se2U obtained in the geranylation and selenation reactions of tRNA catalyzed by MBP-SelU synthase (R5S2U-$tRNA_{Lys}$ subjected to geranylation and selenation reactions).

Synthetic Procedures

***Synthesis of a R5-substituted 2-oxo-, 2-thio-, S-geranyl-2-thio- and 2-seleno-uridines*:** All nucleoside standards for LC-MS analysis were synthesized at Lodz University of Technology. Procedures of synthesis of nm5U, mnm5U, cmnm5U, s2U, nm5S2U, mnm5S2U, cmnm5S2U, Se2U, mnm5Se2U, cmnm5Se2U, ge2U, mnm5geS2U, cmnm5geS2U were previously described [14,17,31-33].

Chemical synthesis of nm5Se2U, nm5geS2U

General Remarks

Thin layer chromatography was done on silica gel coated plates (60F254, Merck), and Merck silica gel 60 (mesh 230–400, Merck) was used for column chromatography. NMR spectra were recorded at a 700 MHz (for ^{1}H) instrument and at 176 MHz for ^{13}C. Chemical shifts (δ) are reported in ppm relative to TMS (an internal standard) for ^{1}H and ^{13}C. The signal multiplicities are described as s (singlet), d (doublet), dd (doublet of doublets), t (triplet), q (quartet), m (multiplet), and bs (broad singlet). High-resolution mass spectrometry (HRMS) measurements were performed using Synapt G2Si mass spectrometer (Waters) equipped with an ESI source and quadrupole-Time-of-flight mass analyser.

Synthesis of 5-aminomethyl-S-geranyl-2-thiouridine (nm5Sge2U)

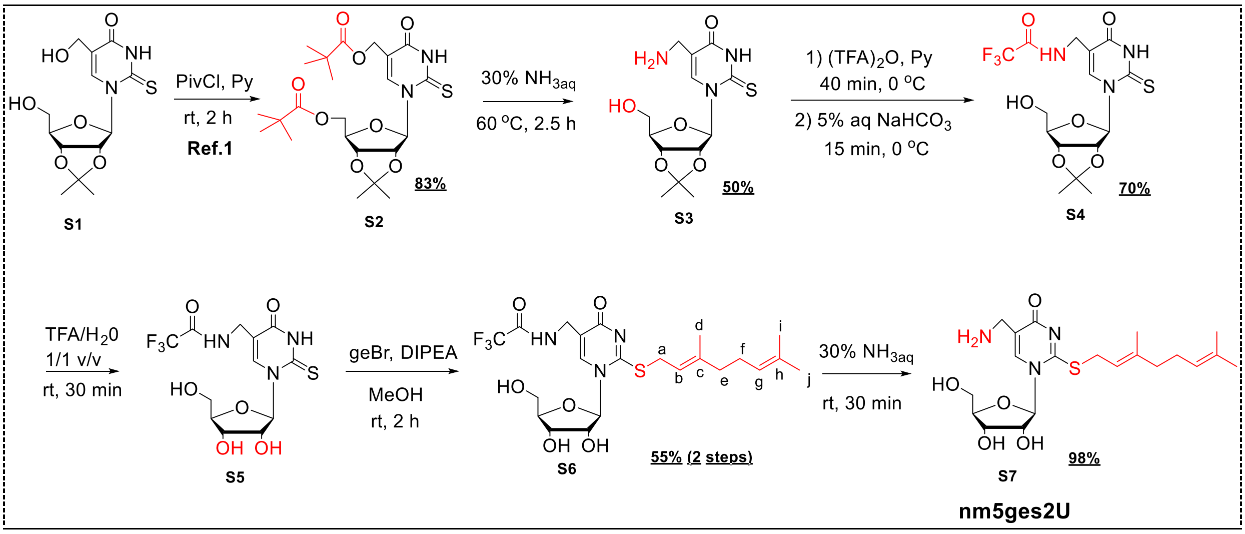

Scheme S1 Synthetic pathway of 5-aminomethyl-*S*-geranyl-2-thiouridine

Synthesis of 2',3'-O-isopropylidene-5-aminomethyl-2-thiouridine (S3)

5-Hydoxymethyl-2-thiouridine **S2** (990 mg, 2.0 mmol, 1 equiv) was treated with 30 % aq NH_3 (60 mL) and incubated in round-bottom flask sealed with rubber septum at 60 °C. After 2 h the traces of undesired product of hydrolysis (2',3'-*O*-isopropylidene-5-hydroxymethyl-2-thiourydine) was indicated by TLC analysis ($CHCl_3$: MeOH, 9:1, v/v). The reaction was stopped, cooled to rt and concentrated under reduced pressure. The crude product **S3** was purified on silica gel column with 10% MeOH in $CHCl_3$ as an eluent affording 2',3'-*O*-isopropylidene-5-aminomethyl-2-thiouridine **S3** in 30 % yield (190 mg). In addition to the main **S3** product, also unreacted substrate **S2** and traces of 2',3'-*O*-isopropylidene-5-aminomethyl-5'-*O*-pivaloyl-2-thiouridine and 2',3'-*O*-isopropylidene-5-hydroxymethyl-2-thiouridine were obtained. **TLC:** R_f= 0.09 ($CHCl_3$: MeOH, 9:1, v/v). **^{1}H NMR** (700 MHz, CD_3OD) δ (ppm): 1.32 (s, 3H, CH_3C<), 1.54 (s, 3H, CH_3C<), 3.57 (s, 2H, CH_2-5,1), 3.73-3.75 (dd, 1H, ^3J=2.80 Hz, ^2J=11.90 Hz, H5''), 3.84-3.86 (dd, 1H, ^3J=2.10 Hz, ^2J=11.90 Hz, H5'), 4.18-4.22 (m, 1H, H4'), 4.75-4.76 (dd, 1H, ^3J=2.80 Hz, ^3J=6.30 Hz, H3'), 4.81-4.83 (dd, 1H, ^3J=3.75 Hz, ^3J=5.60 Hz, H2'), 6.92-6.93 (d, 1H, ^3J=3.50 Hz, H1'), 8.08 (s, 1H, H6). **^{13}C NMR** (176 MHz, CD_3OD) δ (ppm): 25.61 (-CH_3C<), 27.5 (-CH_3C<), 39.04 (CH_2-5,1), 62.18 (C5'), 80.84 (C3'), 86.53 (C2'), 87.84 (C4'), 94.87 (C1'), 115.21 (C5), 117.89 (>C(CH_3)$_2$), 140.01 (C6), 162.62 (C4), 177.61 (C2). HRMS calcd for $C_{13}H_{18}N_3O_5S$ [M-H]$^-$ 328.0967, found 328.0966; HRMS calcd for $C_{13}H_{19}N_3O_5SCl$ [M+Cl]$^-$ 364.0735, found 364.0734.

Synthesis of *2',3'-O-isopropylidene-5-(N-trifluoroacetyl)aminomethyl-2-thiouridine (S4)*

Nucleoside **S3** (190 mg, 0.58 mmol, 1 equiv) was dissolved in anhydrous pyridine (13 mL), cooled in an ice bath, and trifluoroacetic anhydride (TFA_2O, 586 µL, 2.9 mmol, 5 equiv) was added dropwise. The mixture was stirred at rt for 40 min. The reaction was cooled to 0 °C, and quenched with 5% aq. $NaHCO_3$ (390 µL). After 10 min the solution was extracted with $CHCl_3$ (3 x 1.7 mL). The combined organic layers were dried over $MgSO_4$, filtered and concentrated under reduced pressure. Residual pyridine was removed by co-evaporation with anhydrous toluene. The resulting foam was purified by column chromatography with 3% MeOH in $CHCl_3$ as an eluent affording product **S4** in 70% yield (166 mg). **TLC:** R_f=0.7 ($CHCl_3$: MeOH, 85:15 v/v). **^{1}H NMR** (700 MHz, $CDCl_3$) δ (ppm): 1.38 (s, 3H, CH_3C<), 1.61 (s, 3H, CH_3C<), 3.85-3.87 (dd, 1H, ^3J=2.80 Hz, ^2J=11.90 Hz, H5''), 4.07-4.09 (dd, 1H, ^3J=2.1 Hz, ^2J=12.60 Hz, H5'), 4.16-4.23 (m, 2H, CH_2-5,1), 4.46-4.47 (m, 1H, H4'), 4.82-4.83 (dd, 1H, ^3J=2.10 Hz, ^2J=5.60 Hz, H2'), 4.89-4.90 (dd, 1H, ^3J=2.80 Hz, ^2J=5.60 Hz, H3'), 6.64 (d, 1H, ^3J=2.10 Hz, H1'), 8.34 (s, 1H, H6), 10.25 (bs, 1H, NH). **^{13}C NMR** (176 MHz, $CDCl_3$) δ (ppm): 25.42 (-CH_3C<), 27.25 (-CH_3C<), 37.20 (CH_2-5,1), 62.31 (C5'), 80.15 (C3'), 86.36 (C2'), 87.96 (C4'), 96.25 (C1'), 112.76 (>C(CH_3)$_2$), 114.01 (C5), 113.25-118.14 (q, CF_3, ^2J=286.88 Hz), 141.63 (C6), 158.14-158.44 (q, C(O)CF_3, ^1J=38.72 Hz), 160.44 (C4), 175.22 (C2). HRMS calcd for $C_{15}H_{17}N_3O_6SF_3$ [M-H]$^-$ 424.0790, found 424.0795. HRMS calcd for $C_{15}H_{18}N_3O_6SF_3Cl$ [M+Cl]$^-$ 460.0557, found 460.0559.

Synthesis of *5-(N-trifluoroacetyl)aminomethyl-S-geranyl-2-thiouridine (S6)*

Nucleoside **S4** (50 mg, 0.1 mmol, 1 equiv) was treated with 50% aq trifluoroacetic acid (TFA, 290 µL). After being stirred for 45 min at rt, anhydrous toluene (300 µL) was added and the mixture was concentrated under reduced pressure. The solid residue was co-evaporated with anhydrous toluene affording 36 mg of crude product **S5** (**TLC:** R_f= 0.56, $CHCl_3$:MeOH, 8:2, v/v). Then, compound **S5** (36 mg, 0.09 mmol, 1 equiv) was dissolved in anhydrous MeOH (1 mL) and *N,N*-diisopropylethylamine (DIPEA, 72 µL, 0.42 mmol, 4.5 equiv), and geranyl bromide (geBr, 81 µl, 0.42 mmol, 5.5 equiv) were added. After stirring at rt for 10 h the mixture was concentrated under reduced pressure. The residue was dissolved in ethyl acetate and washed with water. Water layer was separated and extracted twice with ethyl acetate. The organic layers were combined, dried over $MgSO_4$ and concentrated under reduced pressure. The solid residue was purified by column chromatography using 4% MeOH in $CHCl_3$ as an eluent. Compound **S6** was obtained as a white solid in yield 70% over two steps (36 mg). **TLC:** R_f=0.56 ($CHCl_3$: MeOH, 90:10, v/v). **^{1}H NMR** (700 MHz, $CDCl_3$) δ (ppm): 1.62 (s, 3H, H_j), 1.70 (s, 3H, H_j), 1.74 (s, 3H, H_d), 2.04-2.06 (t, 2H, ^3J=8.4 Hz, H_e), 2.09-2.12 (q, 2H, ^3J=7.7 Hz, H_f), 3.87-3.92 (m, 2H, 2xH_a), 3.95-3.98 (m, 1H, H5''), 4.04-4.06 (dd, 1H, H5'), 4.17-4.24 (m, 3H, H4', CH_2-5), 4.38-4.39 (t, 1H, ^3J=4.9 Hz, H3'), 4.42-4.43 (t, 1H, ^3J=4.9 Hz, H2'), 5.07-5.08 (t, 1H, ^3J=6.3 Hz, H_g), 5.30-5.32 (t, 1H, ^3J=7.7 Hz, H_b), 5.94 (d, 1H, ^3J=4.2 Hz, H1'), 8.07-8.09 (t, 1H, ^3J=5.6 Hz, NH), 8.39 (s, 1H, H6). **^{13}C NMR** (176 MHz, $CDCl_3$) δ (ppm): 16.40 (C_d), 17.69 (C_j), 25.65 (C_i), 26.35 (C_f), 31.43 (C_a), 37.66 (C5,1), 39.6 (C_e), 61.31 (C5'), 70.14 (C3'), 75.85 (C2'), 85.48 (C4'), 92.21 (C1'), 113.28-118.18 (q, ^1J=287.23 Hz, $COCF_3$), 115.61 (C_b), 115.87 (C5), 123.59 (C_g), 131.96 (C_h), 138.99 (C6), 143.63 (C_c), 157.58-158.21 (q, ^2J=110.88 Hz, $COCF_3$), 164.20 (C2), 168.97 (C4). HRMS calcd for $C_{22}H_{30}N_3O_6SF_3$ $[M-H]^-$ 520.1729, found 520.1737.

Synthesis of *5-aminomethyl-S-geranyl-2-thiouridine (S7)*

Nucleoside **S6** (33 mg, 0.06 mmol) was treated with 30% NH_{3aq} (2 mL). After stirring at rt for 1h the mixture was concentrated under reduced pressure. The residue was purified by column chromatography using 40% MeOH in $CHCl_3$ as an eluent. The final product **S7** was obtained in 60% yield (15 mg). **TLC:** R_f=0.08 ($CHCl_3$: MeOH, 85:15 v/v). **^{1}H NMR** (700 MHz, $CDCl_3$) δ (ppm): 1.64 (s, 3H, H_j), 1.71 (s, 3H, H_i), 1.81 (s, 3H, H_d), 2.08-2.10 (m, 2H, 2xH_e), 2.13-2.15 (m, 2H, 2xH_f), 3.80-3.81 (m, 2H, H-5,1), 3.84-3.86 (dd, 1H, J=2.1 Hz, J=10.5 Hz, H5''), 3.94-4.02 (m, 3H, 2xH_a, H5'), 4.13-4.14 (m, 1H, H4'), 4.23-4.25 (m, 2H, H2', H3'), 5.11-5.13 (m, 1H, H_g), 5.43-5.46 (m, 1H, H_b), 5.93-5.94 (d, J=3.5 Hz, H1'), 8.53 (s, 1H, H6). **^{13}C NMR** (176 MHz, $CDCl_3$) δ (ppm): 15.07 (C_j), 16.35 (C_d), 24.45 (C_i), 26.01 (C_h), 30.29 (C_a), 38.18 (C-5,1), 39.22 (C_e), 60.35 (C5'), 69.56 (C3'), 75.65 (C2'), 85.83 (C4'), 92.51 (C1'), 116.78 (C5), 116.78 (C_b), 123.46 (C_g), 131.29 (C_h), 138.78 (C6), 142.34 (C_c), 164.15 (C2), 169.08 (C4). **HRMS** calcd. for $C_{20}H_{31}N_3O_5S$ $[M+H]^+$ 426.2063, found 426.2062.

Synthesis of *5-aminomethyl-2-selenouridine (nm5Se2U)*

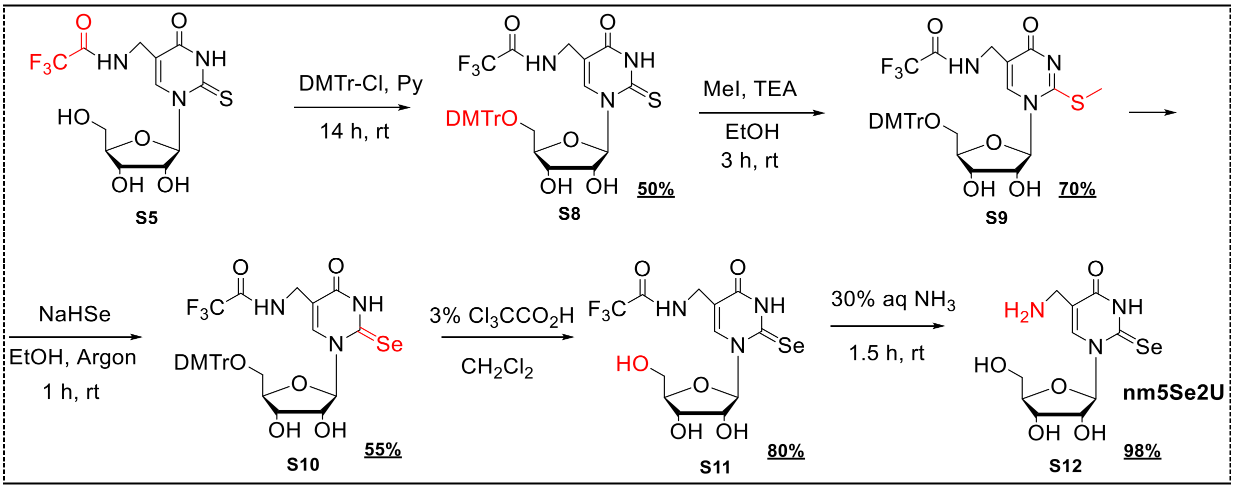

Scheme S2 Synthetic pathway of 5-aminomethyl-2-selenouridine

Synthesis of *5'-O-(4,4'-dimethoxytrityl)-5-(N-trifluoroacetyl)aminomethyl-2-thiouridine (S8)*

Nucleoside **S5** (109 mg, 0.28 mmol, 1 equiv) was dissolved in anhydrous pyridine (2 mL) and 4,4'-dimethoxytrityl chloride (DMTr-Cl, 115 mg, 0.34 mmol, 1.2 equiv) was added. After stirring at rt for 12 h, the starting material **S5** was still not consumed (TLC analysis, $CHCl_3$: MeOH, 90:10, v/v). The next portion of DMTr-Cl (115 mg, 1.2 equiv) was added. After 2 h, the reaction was quenched with water (1.2 mL) and extracted with $CHCl_3$ (3 x 3.5 mL). The combined organic layers were dried over $MgSO_4$ and concentrated under reduced pressure. Pyridine was removed by co-evaporation with anhydrous toluene. 5'-DMTr-2-thiouridine **S8** was purified by column chromatography using 1% MeOH in $CHCl_3$ as an eluent. Product **S8** was obtained in 50% yield (100 mg) as a mixture of rotamers about –NC(O)CF_3 amide bond (two chemical shifts were observed for some ^{13}C NMR resonanses; secondary shifts in ^{13}C NMR spectrum are given in parentheses). **TLC:** R_f=0.63 ($CHCl_3$: MeOH, 90:10, v/v). **^{1}H NMR** (700 MHz, $CDCl_3$) δ (ppm): 3.51-3.54 (m, 4H, CH_2-5,1, H5', H5''), 3.79 (s, 6H, 2xOCH_3), 4.23-4.25 (m, 1H, H4'), 4.42-4.47 (m, 2H, H2', H3'), 6.35 (s, 1H, H1'), 6.85-6.88 (m, 4H, H_{Ar}), 7.23-7.36 (m, 9H, H_{Ar}), 8.14 (s, 1H, H6). **^{13}C NMR** (176 MHz, $CDCl_3$) δ (ppm): 37.06 (CH_2-5,1), 55.39 (2xOCH_3), 62.00 (C5'), 69.95 (C3'), 76.09 (C2'), 84.49 (C4'), 87.25 (-C-Ph_3), 94.56 (C1), 113.62 (C_{Ar}), 113.96 (C5), 113.62-118.19 (q,^1J=287.4 Hz, COCF_3), 127.31 (C_{Ar}), 128.27 (C_{Ar}), 130.33 (C_{Ar}), 130.36 (C_{Ar}), 135.26 (C_{Ar}), 135.55 (C_{Ar}), 139.81 (C6), 144.54 (C_{Ar}), 156.81-157.45 (q, ^2J =37.1 Hz, COCF_3), 158.91 (C_{Ar}), 160.46 (C4), 175.13 (C2). **HRMS** calcd. for $C_{33}H_{32}N_3O_8SF_3$ $[M-H]^-$ 686.1784, found 686.1780.

Synthesis of *5'-O-(4,4'-dimethoxytrityl)-5-(N-trifluoroacetyl)aminomethyl-S-methyl-2-thiouridine (S9)*

Nucleoside **S8** (52 mg, 0.076 mmol, 1 equiv) was dissolved in anhydrous ethanol (0.8 mL) and anhydrous triethylamine (TEA, 32 µL, 0.088 mmol, 3 equiv), and methyl iodide (MeI, 14 µL, 0.088 mmol, 3 equiv) were added. After stirring at rt for 3 h the mixture was concentrated under reduced pressure. The residue was dissolved in CH_2Cl_2 (2.5 mL) and washed with water (1.3 mL). Water was extracted with CH_2Cl_2 (2 x 2.5 ml). The organic layers were combined, dried over $MgSO_4$ and concentrated under reduced pressure. The solid residue was co-evaporated with anhydrous toluene and purified by column chromatography using 12% MeOH in methylene chloride as an eluent. *S*-Methyl-2-thiouridine **S9** was obtained in 70% yield (36 mg) as a mixture of rotamers about –NC(O)CF_3 amide bond (two chemical shifts were observed for some ^{13}C NMR resonanses; secondary shifts in ^{13}C NMR spectrum are given in parentheses).. **TLC:** R_f=0.11 (CH_2Cl_2 : acetone, 85:15, v/v). **^{1}H NMR** (700 MHz, $CDCl_3$) δ (ppm): 2.60 (s, 3H, SCH_3), 3.36-3.51 (m, 4H, H5'', H5', CH_2-5,1), 3.79 (s, 6H, 2xOCH_3), 4.27-4.28 (m, 1H, ^3J=2.8 Hz, H4'), 4.52-4.53 (m, 1H, H3'), 4.59-4.60 (m, 1H, H2'), 5.91-5.92 (d, 1H, ^3J=6.3 Hz, H1'), 6.87-6.88 (d, 4H, ^3J=9.1 Hz; H_{Ar}), 7.32-7.44 (m, 9H, H_{Ar}), 7.90 (s, 1H; H6). **^{13}C NMR** (176 MHz, $CDCl_3$) δ (ppm): 15.42 (SCH_3), 37.72 (CH_2-5,1), 55.35 (2xOCH_3), 63.35 (C5'), 71.67 (C3'), 75.42 (C2'), 84.90 (C4'), 87.51 (-C-Ph_3), 92.12 (C1), 113.58 (C_{Ar}), 115.82 (C5), 113.19-118.26 (q, ^1J=151.4 Hz, COCF_3), 127.38 (C_{Ar}), 128.26 (C_{Ar}), 128.39 (C_{Ar}), 130.36 (C_{Ar}), 130.45 (C_{Ar}), 135.04 (C_{Ar}), 135.29 (C_{Ar}), 138.22 (C6), 144.20 (C_{Ar}), 156.46-156.99 (q, ^2J =37.3 Hz, COCF_3), 158.96 (C_{Ar}), 164.95 (C2), 168.87 (C4). HRMS calcd. for $C_{34}H_{34}N_3O_8SF_3$ $[M+H]^+$ 702.2097, found 702.2103.

Synthesis of *5'-O-(4,4'-dimethoxytrityl)-5-(N-trifluoroacetyl)aminomethyl-2-selenouridine (S10)*

Selenium (32 mg, 0.4 mol, 10 equiv) was suspended in anhydrous ethanol (0.3 mL) at 0 ^{o}C and then sodium borohydride ($NaBH_4$, 23 mg, 0.6 mol, 15 equiv) was added. The reaction was stirred for 2 h at 0 ^{o}C to get the clear solution of ethanolic NaSeH. Then *S*-methyl-2-thiouridine **S9** (30 mg, 0.04 mmol, 1 equiv) was added and the mixture was stirred for 1 h at room temperature. The reaction mixture was then evaporated under reduced pressure. The residue was dissolved in the mixture of ethyl acetate – water (6 mL, 1:1, v/v). The organic phase was separated and water was extracted several times with ethyl acetate (6 x 5 mL). Organic layers were combined, dried over anhydrous $MgSO_4$ and evaporated under reduced pressure. The residue was purified by flash column chromatography using argon overpressure and 5% MeOH in CH_2Cl_2 as an eluent. Pure selenouridine derivative **S10** was obtained in 55 % yield (20 mg) as a mixture of rotamers about –NC(O)CF_3 amide bond (two chemical shifts were observed for some ^{1}H and ^{13}C NMR resonanses; secondary shifts in NMR spectra are given in parentheses). **TLC:** R_f=0.26 ($CHCl_3$: MeOH, 95:5, v/v). **^{1}H NMR** (700 MHz, $CDCl_3$) δ (ppm): 3.49-3.50 (d, 2H, CH_2-5,1), 3.56-3.60 (m, 2H, H5', H5''), 3.81 (s, 6H, 2xOCH_3), 4.27-4.28 (m, 1H, H4'), 4.48-4.50 (m, 1H, H2'), 4.55-4.56 (dd, 1H, H3'), 6.45 (d, 1H, H1'), 6.88-6.90 (m, 4H, H_{Ar}), 7.29-7.38 (m, 9H, H_{Ar}), 8.19 (s, 1H, H6). **^{13}C NMR** (176 MHz, $CDCl_3$) δ (ppm): 36.97 (CH_2-5,1), 55.24 (2xOCH_3), 61.60 (C5'), 69.46 (C3'), 76.24 (C2'), 84.70(C4'), 87.11 (-CPh_3), 96.79 (C1), 113.19 (C_{Ar}), 113.48 (C_{Ar}), 115.33 (C5), 114.71-116.35 (q, ^1J=575.7 Hz, COCF_3), 127.17 (C_{Ar}), 127.78 (C_{Ar}), 128.14 (C_{Ar}), 129.14 (C_{Ar}),

130.22 (C_{Ar}), 135.04 (C_{Ar}), 135.37 (C_{Ar}), 136.18 (C_{Ar}), 139.30 (C6), 156.52-157.16 (q, ^{2}J=75.7 Hz, $COCF_3$), 158.82 (C_{Ar}), 158.79 (C_{Ar}), 159.18 (C4), 175.37 (C2). HRMS calcd. for $C_{33}H_{32}N_3O_8F_3Se$ $[M+H]^+$ 736.1385, found 736.1399.

Synthesis of *5-aminomethyl-2-selenouridine (S12)*

The solution of **S10** (18 mg, 0.03 mmol, 1 equiv) in CH_2Cl_2 was introduced to silica gel placed in *flash* column. Then, the solution of 3% Cl_3CCO_2H in CH_2Cl_2 (2 mL) was passed through the column using argon overpressure. The elution was continued with 2% MeOH in CH_2Cl_2 using argon overpressure. Nucleoside **S11** was obtained in 80% yield (8 mg). **TLC** R_f=0.67 (isopropanol:ammonia:water, 7:2:1, v:v:v). **^{1}H NMR** (250 MHz, CD_3OD) δ (ppm): 3.76-3.82 (dd, 1H, ^{2}J=12.5 Hz, H5''), 3.90-3.96 (dd, 1H, ^{2}J=12.5 Hz, H5'), 4.05-4.14 (m, 4H, CH_2-5,1, H3', H4'), 4.22-4.25 (dd, 1H, ^{3}J=5.0 Hz, ^{3}J=2.5 Hz, H2'), 6.70-6.71 (d, 1H, *J*=2.5 Hz, H1'), 8.45 (s, 1H, H6). Compound **S11** (8 mg, 0.02 mmol, 1 equiv) was treated with $30\%_{aq}$ NH_3 (0,8 mL). After stirring at rt for 1.5 h the reaction mixture was concentrated under reduced pressure. The solid residue was purified by column chromatography using 30% MeOH in $CHCl_3$ as an eluent. Compound **S12** was obtained in 98% yield (6 mg). **TLC:** R_f=0.35 (isopropanol:ammonia:water, 7:2:1, v:v:v). **^{1}H NMR** (700 MHz, D_2O) δ (ppm): 3.92-3.94 (m, 3H, CH_2-5,1, H5''), 4.06-4.08 (dd, 1H, ^{2}J=2.8 Hz, ^{3}J=13.3 Hz, H5'), 4.22-4.24 (m, 1H, H4'), 4.26-4.28 (^{2}J=4.9 Hz, ^{3}J=7.0 Hz, H3'), 4.42-4.43 (dd, 1H, ^{2}J=2.8 Hz, ^{3}J=4.9 Hz, H2'), 6.92-6.93 (d, 1H, ^{2}J=2.7 Hz, H1'), 8.29 (s, 1H, H6). **^{13}C NMR** (176 MHz, D_2O) δ (ppm): 37.87 (C-5,1), 59.90 (C5'), 68.39 (C3'), 75.22 (C2'), 83.83 (C4'), 95.96 (C1'), 112.00 (C5), 140.58 (C6), 168.08 (C4), 175.64 (C2). HRMS calcd. for $C_{10}H_{16}N_3O_5Se$ $[M+H]^+$ 338.0255, found 338.0257.

Synthesis of S2U and geS2U phosphoroamidites*:* The phosphoramidite derivatives of S2U and geS2U were prepared in-house according to the previously described procedures [11,20,33].

5'-O-(4,4'-Dimethoxytrityl)-2'-O-(tert-butyldimethylsilyl)-2-thiouridine-3'-(cyanoethyl N,N-diiso-propyl phosphoramidite: The first step was the silylation of 2-thiouracil (2.00 g, 15.6 mmol) by treatment with trimethylsilyl chloride (TMSCl) (10.17 g, 37.4 mmol, 2.4 equiv.) in the presence of trimethylamine (3.78 g, 37.4 mmol, 2.4 equiv.). The reaction was carried out under reflux and the crude product was purified by evaporation of excess of TMSCl and TEA in vacuum. Next, the obtained bis-silyl derivative of 2-thiouracil (3.64 g, 13.38 mmol, 1.1 equiv.) was reacted with 1-*O*-acetyl-2,3,5-tri-O-benzoyl-β-D-ribofuranose (6.08 g, 12 mmol) in DCE followed by the addition of tin (IV) chloride (4.49 g, 17.25 mmol, 1.4 equiv.) at -5 °C. and the reaction was continued at room temperature overnight. The reaction was quenched by 5 % aqueous sodium bicarbonate treatment and the crude 2-thiouridine derivative was purified by column chromatography. The hydroxyl protecting group in *1-(2',3',5'-tri-O-benzoyl-beta-D-*ribofuranosyl)-2-thio-uridine were then removed with sodium methoxide in methanol and neutralized by Dowex 50wx8 H^+ form. In the next step the 5' hydroxyl of 2-thiouridine (1.23 g, 4.74 mmol) was protected by dimethoxytrityl group in reaction with DMT-Cl (1.80 g, 5.21 mmol, 1.12 equiv.) to yield 5'-*O*-DMT-2-thiouridine (2.44 g, 4.32 mmol). The latter one was reacted with TBDMS-Cl (0.98g, 6.51 mmol, 1.5 equiv.) in pyridine in the presence of imidazole (1.18 g, 17.28 mmol, 4 equiv.). As a result, the mixture of corresponding 2'-O-TBDMS and 3'-*O*-TBDMS derivatives was obtained. The 2' and 3' isomers were then separated by silica gel chromatography and the desired 2'-*O*-TBMD-5'-*O*-DMTr 2-thiouridine (1.1 6g, 1.72 mmol) was phosphitylated with cyanoethyl-N,N-diisopropylchlorophosphoramidite (0.81 g, 3.44 mmol, 2 equiv.) in the presence of DIPEA (0.45 g, 3.44 mmol, 2 equiv.) under argon atmosphere. The final 5'-*O*-(4,4'-dimethoxytrityl)-2'-*O*-(tert-butyldimethylsilyl)-2-thiouridine-3'-(cyanoethyl-N,N-diiso-propylphosphoramidite) was purified by silica gel chromatography to yield 1.13 g of the pure compound in form of white foam [1.29 mmol, 75%, HRMS (ESI-TOF): molecular formula, $C_{36}H_{44}N_2O_7SSi$; $[M-H]^-$: 875.3659 (calc. 875.3639), ^{31}P NMR (200 MHz, CD_3CN) δ 150.05, 150.01 ppm].

***5'-O-(4,4'-Dimethoxytrityl)- 2'-O-(tert-Butyldimethylsilyl)- S-geranyl-2-thiouridine-3'-(cyanoethyl N,N-diisopropylphosphoramidite*:** 5'-*O*-DMTr-2'-TBDMS-2-S2U phosphoramidite (65.00 mg, 0.074 mmol) was dissolved in anhydrous methanol (dried over 3A molecular sieves, 3 mL) under argon atmosphere. Then the mixture of geranyl bromide (32.22 mg, 0.148 mmol, 2 equiv.) and diisopropylethylamine (38.36 mg, 0.297 mmol, 4 equiv.) was added and the mixture was stirred at room temperature (rt) for 3 h (monitored by TLC plates). Then the solvent was removed in vacuo and the crude product was isolated by silica gel column chromatography (230–400 mesh, chloroform : methanol 50:1 (v/v)) to yield a desired phosphoramidite as a white foam (73.60 mg, 0.073 mmol, 98%). ^{31}P NMR (200MHz, CD_3CN) δ 150.93, 148.53 ESI-MS $[M+H]^+$: 1013.5362 (calc. 1013.5048).

Synthesis of model RNA oligonucleotides*:* The RNA oligomers of the 5'-GUUGACU**X**UUAAUCAAC -3' sequence, where X is S2U or geS2U, were synthesized automatically on a 0.2 µmole scale with the use of an H6 GeneWorld DNA/ RNA automated synthesizer (K&A, Laborgeraete GbR, Schaafheim, Germany), according to already published procedure [11,20]. The commercially available phosphoramidites of A, C, U, and G (Glen Research) protected at the 5'- and 2'-hydroxyl functions with DMTr and TBDMS groups respectively, were used. The exocyclic amine functions were masked with phenoxyacetyl (A and G units), or acetyl (C) groups (Proligo). The rC(tac)-succinyl-CPG (Proligo) suport and 0.07 M solutions of the monomers in acetonitrile (ACN) were used. The phosphoramidite derivatives of S2U, or geS2U were prepared in-house, as described above. The phosphoramidities (a 10-fold molar excess) were mixed with a BMT activator (0.25 M in ACN) and the coupling was executed for 10 min. For the oxidation step, during the synthesis of the oligomer containing S2U or geS2U, a commercially available 0.02 M solution of I_2 in THF/H_2O/pyridine (2 min) was applied. The capping step was performed with TAC_2O (tert-butylphenoxyacetyl acetic anhydride) in THF using a mixture of Fast deprotection Cap A (Proligo) and Cap B (Proligo) (1:1.1, v/v) for 2 min. After the last coupling the DMTr group was removed and the support was washed, dried and transferred to a screw cap glass vial. Then a TEA/ACN mixture (265 µL, 1:1, v/v) was added and the suspension was stirred for 20 min, and then the volatile components were removed. The support-bound RNA was washed with ACN (4 × 100 µL) and dried *in vacuo* for 30 min. The detritylated S2U-RNA oligomer was cleaved from the support and deprotected at the exoamine functions with aq. NH_3/EtOH (3:1, v/v, 1.5 mL) for 16h at 40 °C. The supernatant was collected and the support was washed with ethanol/H_2O (1:1, v/v, 4 × 150 µL). Whereas, the support linked to geS2U-RNA oligomer was treated with TEA/acetonitrile mixture (265 µL, 1:1, v/v) for 20 min at room temperature. After removal of the volatile components, the dried beds were treated with ethanolic ammonia (8 M, 340 µL) for 8h at room temperature. The supernatant was removed and the support was washed with anhydrous ethanol (4 × 150 µL). In both cases the combined washings were evaporated on a Speed-Vac concentrator. After evaporating the solution containing an appropriate oligomer (S2U-RNA or gerS2U-RNA), the solid residue was treated with TEA, 3HF (Triethylamine trihydrofluoride) (300 µL) for 22-24h, at room temperature. The reaction was quenched by the addition of 10 µL of water and the crude RNA was precipitated (using 1mL of n-butanol) and purified by reversed-phase RP HPLC (Shimadzu, Japan). The fully deprotected RNA was then purified by reversed-phase (RP) HPLC (Shimadzu, Japan) using a Kinetex C18 column (5µ, 100A; 250 x 4.60 mm, Phenomenex). The products were isolated in the gradient of buffer B: 0–5 min 0% B, 5–21 min 0– 100% B, 21–26 min 100% B, 26–31 min 100–0% B, and 31-32 min 0% B (buffer A: 0.1 M CH_3COONH_4; pH 6.8, buffer B: 0.1 M CH_3COONH_4/40 % CH_3CN). The appropriate fractions were collected and desalted using a SepPak C-18 (Waters) cartridge and the identity of the obtained oligomers (S2U-RNA or geS2U-RNA) were confirmed by ESI-MS.

Synthesis of selenophosphate $SePO_3^{3-}$: The synthesis of selenophosphate was done in two steps according to the already described procedures [20,34,35]. Briefly: Tris(trimethylsilyl) phosphite (TCI Chemicals, Japan) (2.1 g, 0.007 mol) was placed in a 10 mL two necked round-bottom flask equipped with a magnetic stirrer, thermometer, and a reflux condenser. The flask was heated to 50 °C and that temperature was kept during the portion wise addition of powered, elemental selenium followed by overnight stirring at 50 °C. The excess selenium was filtered off and vacuum distillation (b.p. 51–52 °C/0.1 mmHg) of the filtrate furnished *O,O,O*-tris(trimethylsilyl)phosphoroselenoate (1.67 g, 0.0044 mol), 63% yield; spectral data: ^{31}P NMR ($CDCl_3$) δ: 21.78 p.p.m. (J(PSe) 924 Hz); ^{1}H NMR ($CDCl_3$) δ: 0.33 ppm (s, 27H, $Si(CH_3)_3$). To remove the trimethylsilyl groups from *O,O,O*-tris(trimethylsilyl)phosphoroselenoate (11.32 mg, 0.03 mmol) in dry chloroform (0.5 mL, the final concentration 60 mM), an equal volume of 0.1 M Tricine-KOH buffer (pH 7.2) containing 20 mM DTT and 60 mM $MgCl_2$ was added. The phases were separated by centrifugation. All experiments were carried out under an inert argon atmosphere. The resultant solution of selenophosphate salt (^{31}P NMR (D_2O) δ: 21.59 ppm) was used in the of enzymatic selenation of geS2U-RNA to Se2U-RNA.

Protein Procedures

Synthesis and purification of MBP-SelU: The bacterial wild type selenouridine tRNA synthase gene (*wtselU*) was previously isolated from total cellular RNA of *E. coli* [11,20]. The insert containing the *wtselU* gene was isolated from the pET28c-SelU plasmid in a PCR reaction with specific primers: Fw: 5'-AAAAAACCATGGAGAGACACAGGGAACAGG-3', Rev: 5'-TGCAGGGAATTCTTACCGCGCCTT AACCCATT-3' and cloned into a pMAL-c5x expression vector (New England BioLabs, NEB), between

Nco I – Eco RI recognition sites. The correct sequence of the gene insert was confirmed by DNA sequencing reaction (CoreLab, Lodz, Poland). The recombinant fusion protein (MBP-wtSelU) was overexpressed in NEB Express, ER2523 *E. coli* cells (NEB) in 2YT broth medium supplemented with ampicillin (100 mg/mL), after induction with 1 mM isopropyl-β-D-thiogalactopyranoside (IPTG). Protein was isolated from bacterial cells and purified in a two-step procedure 1) affinity chromatography on Amylose Resin (New England BioLabs, NEB), according to the protocol of NEB: column buffer: 20 mM Tris-HCl, pH 7.4, 200 mM NaCl, 1 mM EDTA; elution buffer: 20 mM Tris-HCl, pH 7.4, 200 mM NaCl, 1 mM EDTA; 10 mM maltose and 2) gel filtration on Superdex 200 resin (GE Healthcare), column buffer: 20 mM Tris-HCl, pH 7.4; 25 mM NaCl. All steps of protein preparation were performed at 4 °C. Protein purity was analyzed by SDS gel electrophoresis. Enzyme preparations were concentrated to ~70 mg/mL (AMICON ULTRA, cutoff 50 KDa, Merck- Millipore), divided into small aliquots (e.g. 10 µL), frozen in liquid nitrogen and stored at -80 °C. Protein concentration was determined spectrophotometrically using Bradford Reagent (Sigma Aldrich).

Table S1 Sequences and ESI-MS characteristic of the model ASL-RNAs used in studies
Abbreviations used: chs – chemical synthesis; es – enzymatic synthesis; chs/es – chemical or enzymatic synthesis, nd- not determined

A. 17-mers:

Name	RNA sequence (5'→3')	Preparation method	MW [g/mol]	Mass* [Da]
S2U(33)-RNA	GUUGAC**S2U**UUUAAUCAA	chs	5349	5349.6
S2U(34)U-RNA^{Lys}	GUUGACU**S2U**UUAAUCAAC	chs	5349	5348.6
mnm5S2U(34)-RNA^{Lys}	GUUGACU**mnm5S2U**UUAAUCAAC	chs	5391	5391.6
S2U(34)A-RNA	GUUGACU**S2U**AUAAUCAAC	chs	5372	5371.7
S2U(34)G-RNA	GUUGACU**S2U**GUAAUCAAC	chs	5388	5387.7
S2U(34)C-RNA	GUUGACU**S2U**CUAAUCAAC	chs	5347	5347.7
geS2U(34)U-RNA^{Lys}	GUUGACU**geS2U**UUAAUCAAC	es	5485	5484.8
mnmgeS2U(34)U-RNA^{Lys}	GUUGACU**mnm5geS2U**UUAAUCAAC	es	5527	5527.8
geS2U(34)A-RNA	GUUGACU**geS2U**AUAAUCAAC	es	5510	nd
geS2U(34)G-RNA	GUUGACU**geS2U**GUAAUCAAC	es	5524	5523.7
geS2U(34)C-RNA	GUUGACU**geS2U**CUAAUCAAC	es	5483	5483.8
Se2U(34)-RNA^{Lys}	GUUGACU**Se2U**UUAAUCAAC	es	5396	5395.6
mnmSe2U-RNA^{Lys}	GUUGACU**mnm5Se2U**UUAAUCAAC	es	5438	5438.6
Cy3-U(34)-RNA^{Lys}	Cy3-GUUGACU**U**UUAAUCAAC	chs	5838	5838.0
Cy3-S2U(34)-RNA^{Lys}	Cy3-GUUGACU**S2U**UUAAUCAAC	chs	5855	5853.9
Cy3-geS2U(34)-RNA^{Lys}	Cy3-GUUGACU**geS2U**UUAAUCAAC	chs/es	5993	5993.0
Cy3-Se2U(34)-RNA^{Lys}	Cy3-GUUGACU**Se2U**UUAAUCAAC	es	5903	5903.0
S2U(35)-RNA	GUUGACUU**S2U**UAAUCAAC	chs	5349	5348.6
geS2U(35)-RNA	GUUGACUU**geS2U**UAAUCAAC	chs/es	5487	5485.8
S2U(36)-RNA	GUUGACUUU**S2U**AAUCAAC	chs	5349	5348.7
geS2U(36)-RNA	GUUGACUUU**geS2U**AAUCAAC	chs/es	5487	5484.7
S2U(34)-RNA^{Arg}	ACGGCCU**S2U**CUAAGCCGU	chs	5377	5377.7
geS2U(34)-RNA^{Arg}	ACGGCCU**geS2U**CUAAGCCGU	chs/es	5515	5513.9

*the results were obtained after deconvolution of the raw ESI mass spectra using the MaxEnt1 algorithm to a zero-charge state mass.

B. Shorter oligomers tested in current studies as substrates for MBP-SelU

Name	RNA sequence (5'→3')	Preparation method	MW [g/mol]	Mass* [Da]
7-mer S2U-RNA	AC**S2U**UUUA	chs	2142	2142.26
3-mer S2U-RNA	U**S2U**U	chs	871	871.3
nucleoside	S2U	chs	260.05	260.3
nucleoside	mnm5S2U	chs	303.33	302.1
nucleoside	geS2U	chs	396.17	396.5
nucleoside	mnm5geS2U	chs	439.57	438.2
nucleoside	Se2U	chs	306.12	306.0
nucleoside	mnm5Se2U	chs	350.23	349.0

*the results were obtained after deconvolution of the raw ESI mass spectra using the MaxEnt1 algorithm to a zero-charge state mass.

Table S2 Detailed conditions for S2U-RNA geranylation and geS2U-RNA selenation reactions catalyzed by MBP-SelU

A. Geranylation reaction conditions:

Modification position	ASL-S2U-RNA [nmol]	MBP-SelU [nmol]	GePP [nmol]	Reaction volume [µL]	Time [min]	Temperature [°C]
**33**	1.113	0.249	5.56	100	60	37
**34**	1.113	0.249	5.56	100	60	37
**35**	1.113	0.249	5.56	100	60	37
**36**	1.113	0.249	5.56	100	60	37
**Arg**	1.145	0.256	5.73	100	60	37
**7-mer**	4.2	0.249	21	100	60	37
**3-mer**	11	0.498	110	100	60	37
**S2U / mnm5S2U**	50	0.249	250	100	60	37

B. Selenation reaction conditions

Modification position	ASL-geS2U-RNA [nmol]	MBP-SelU [nmol]	$SePO_3^{3-}$ [nmol]	Reaction volume [µL]	Time [min]	Temperature [°C]
**33**	1.113	0.249	20	100	30	37
**34**	1.113	0.249	20	100	30	37
**35**	1.113	0.249	20	100	30	37
**36**	1.113	0.249	20	100	30	37
**Arg**	1.145	0.254	20	100	30	37
**7-mer**	-	-	-	-	-	-
**3-mer**	-	-	-	-	-	-
**geS2U/mnm5geS2U**	50	0.249	20	100	30	37

Figure S1 Overexpression and purification of MBP-SelU, visualization of protein bands in SDS polyacrylamide gel stained with dye Coomassie Brillant Blue.

A. MBP-SelU overexpression in the *E. coli* system, line 1 - protein mass standard; line 2 - bacterial proteins and proteins expressed from the "empty" plasmid pMAL-c5x; line 3 - bacterial proteins and proteins expressed from the pMAL-wtSelU plasmid.

B. MBP-SelU purification, line 1 - protein mass standard; line 2 - bacterial mixture of soluble proteins before the purification process; line 3 – proteins after the first purification step on the Amylose Resin; line 4 and line 5 - the second purification step on the Superdex 200 resin: eluted MBP-SelU fusion protein (line 4), eluted MBP (line 5)

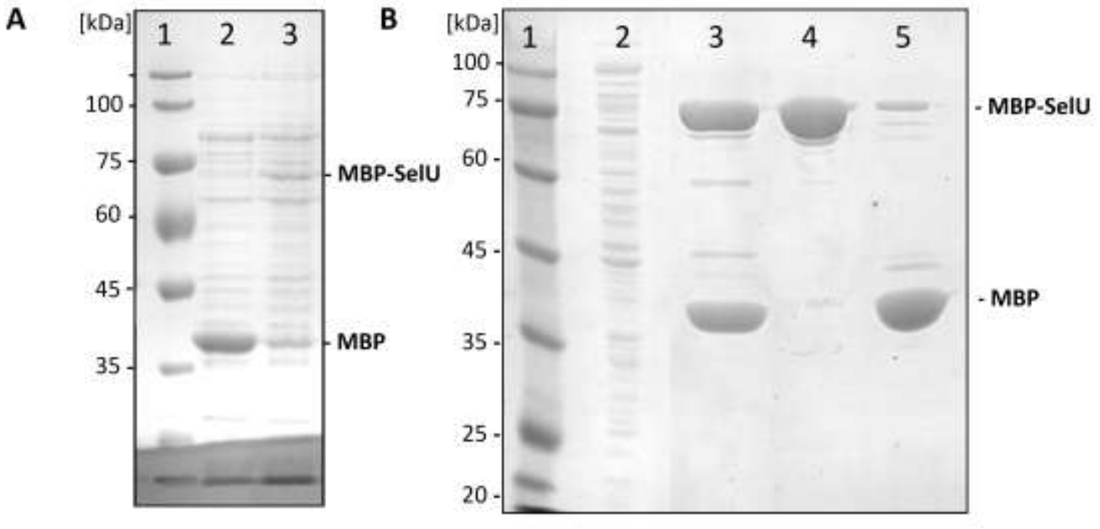

Figure S2 The separation of the pure proteins from the mixture of MBP-SelU and free MBP by gel filtration on the Superdex 200 resin

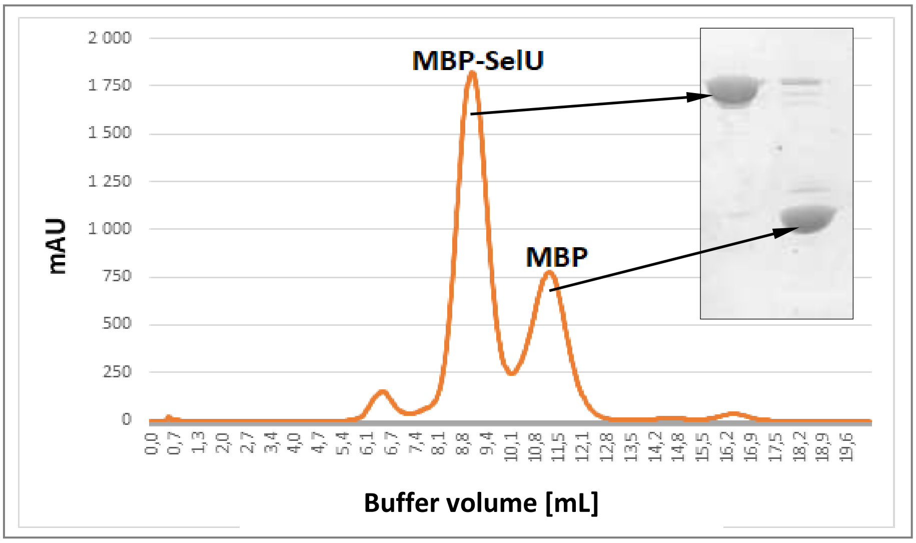

Figure S3 (A-H) ESI-TOF MS analysis of the oligo-RNA standards used in the studies, sequences are listed in the Table S1

A. 17-mer with modification at 33 position, S2U(33)-RNA

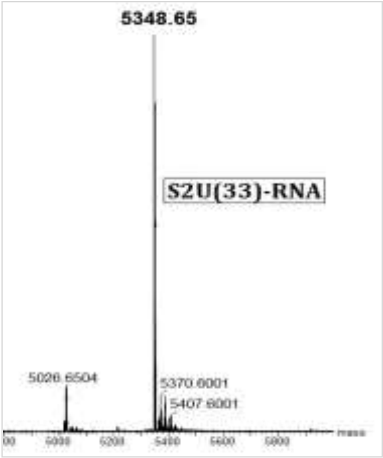

B. 17-mers with modification at 34 position, S2U(34)-RNA, geS2U(34)-RNA, Se2U(34)-RNA

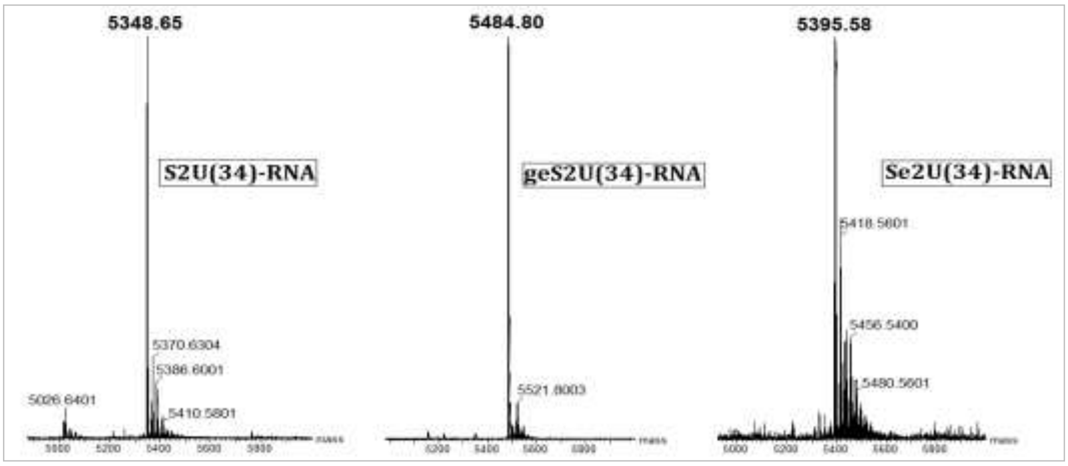

C. 17-mers with modification at 34 position, labeled with Cy3 at the 5'-end

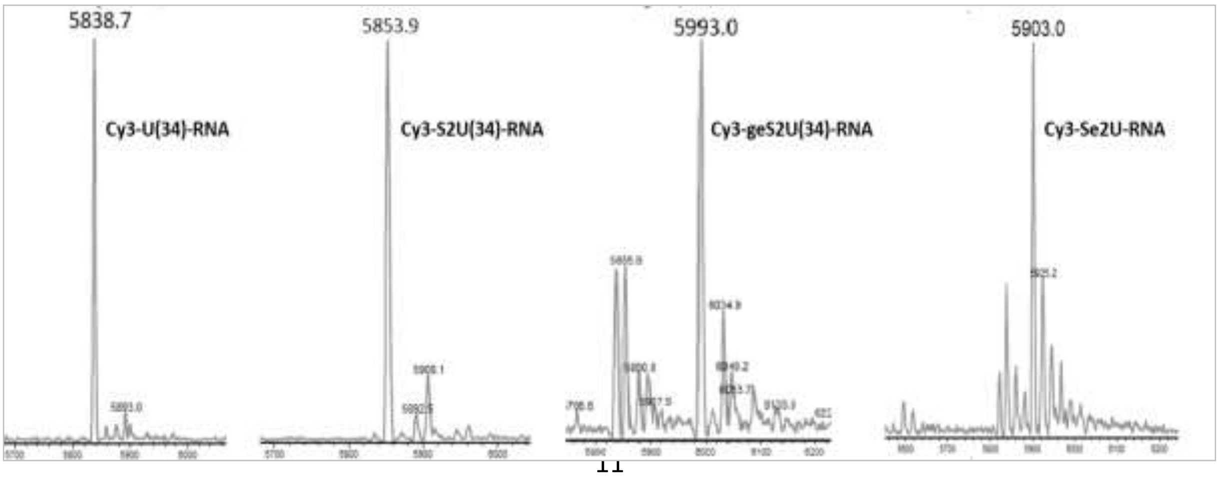

D. 17-mers with modification at 35 position

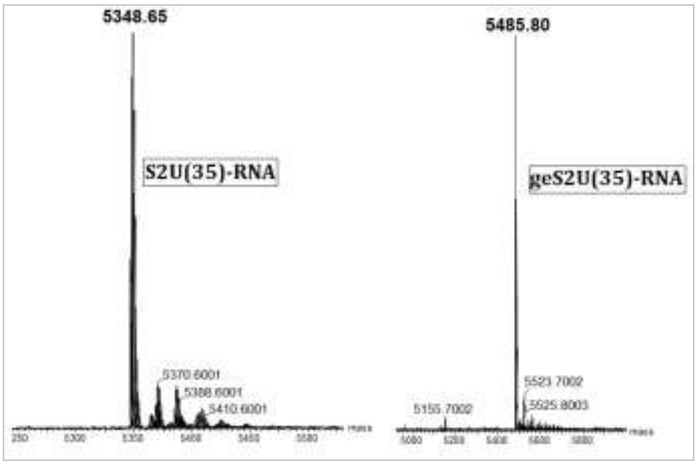

E. 17-mers with modification at 36 position

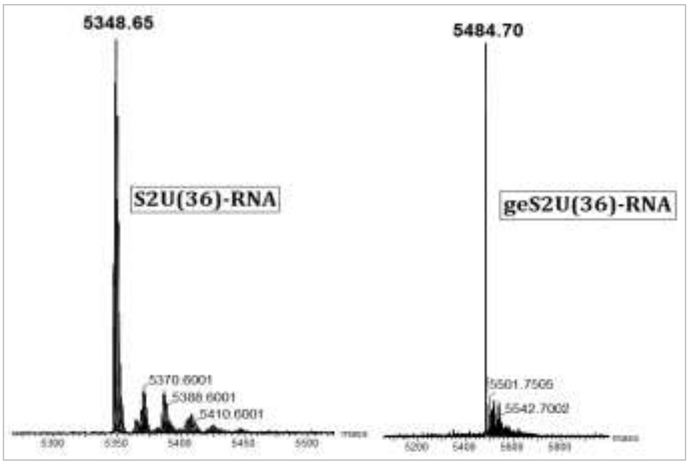

F. 17-mers with Arg ASL sequence

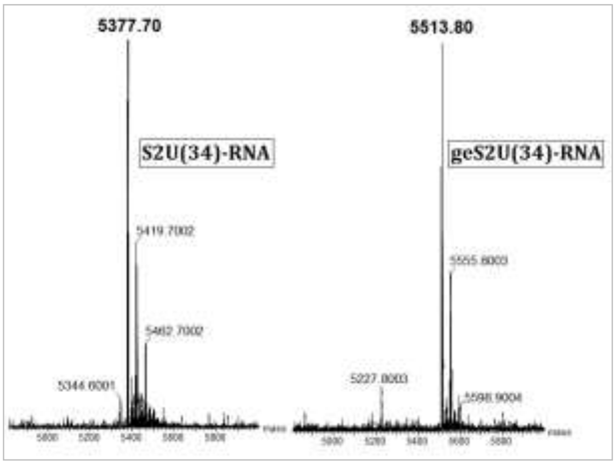

G. 7-mers

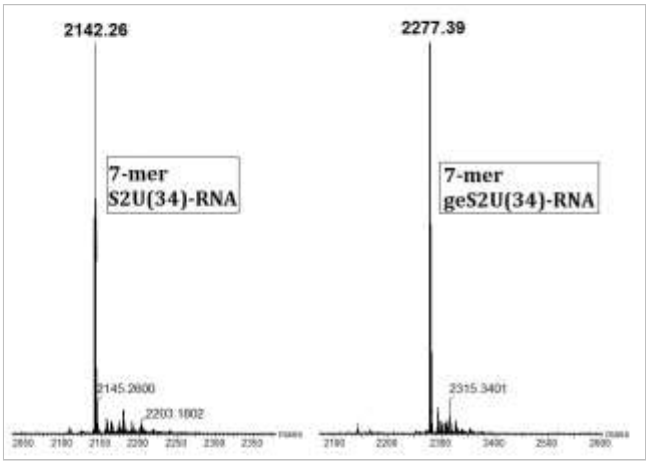

H. 3-mers

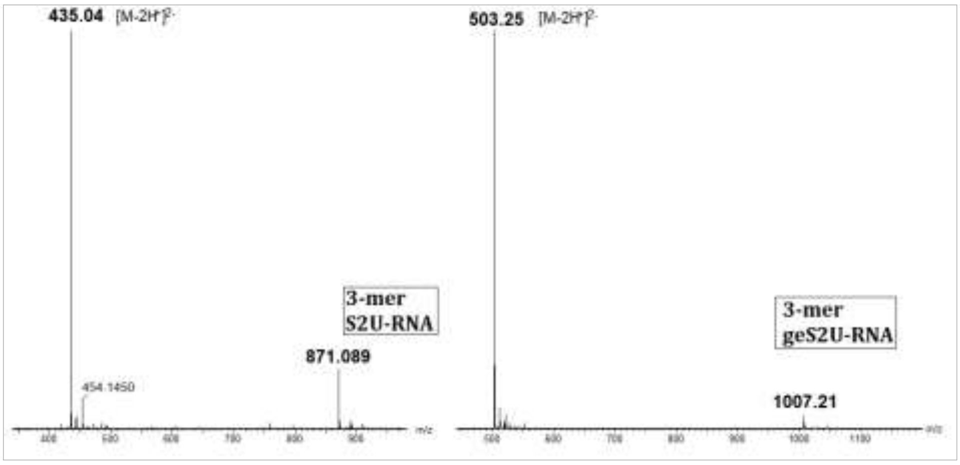

Figure S4 Kinetic data determined for the reactions catalyzed by MBP-SelU

Geranylation of S2U(34)-RNA^{Lys}
k_{cat}= 0.39 ±0.01 [min^{-1}]
K_M= 3.02 ±0.38 [µM]

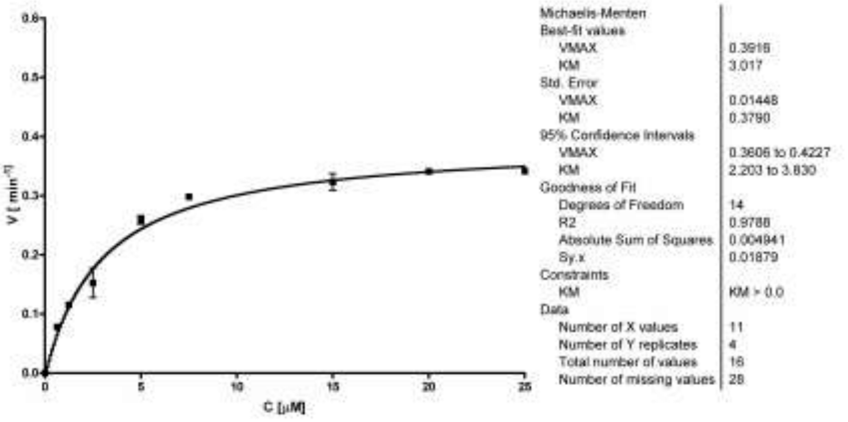

Selenation of geS2U(34)-RNA^{Lys}
k_{cat}=5.07 ±0.13 [min^{-1}]
K_M=0.441 ±0.045 [µM]

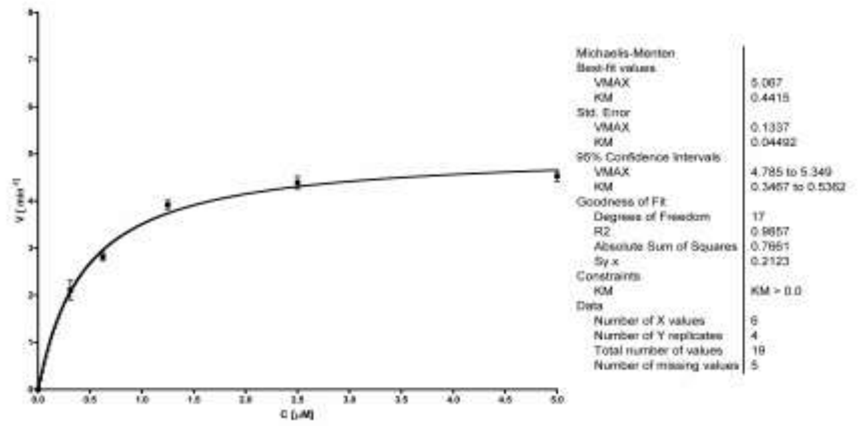

Table S3. Summary of kinetic data of geranylation and selenation reactions catalyzed by MBP-SelU. Reactions were performed with oligonucleotide RNA substrates S2U(34)-RNA^{Lys} (geranylation) and geS2U(34)-RNA^{Lys} (selenation) using MBP-SelU enzyme. For comparison, kinetic data for an identical reaction catalyzed by SelU-His$_6$ are given.

	Geranylation		Selenation	
	**MBP-SelU**	**SelU-His$_6$**	**MBP-SelU**	**SelU-His$_6$**
k_{cat} [min^{-1}]	0.39 ±0.01	0.14 x 10^{-3}	5.07 ±0.13	0.53
K_M [µM]	3.02 ±0.38	1.23 ±0.31	0.441 ±0.045	2.49 ±0.88
k_{cat}/K_M [min^{-1} µM^{-1}]	0.129	0.0001138	11.496	0.2314
Geranylation efficiency (MBP-SelU vs. SelU-His$_6$)	1133	1		
Selenation efficiency (MBP-SelU vs. SelU-His$_6$)			50	1

Figure S5 The chromatographic mobility of the oligo-RNA standards (3-17-mers). Analysis by RP-HPLC (Shimadzu, Japan), Kinetex 5μ C-18 100A; 250 x 4.60 mm column (Phenomenex), buffers (A: 0.1 M CH_3COONH_4; B: 0.1 M CH_3COONH_4/ 40 % CH_3CN)

A. Nucleosides: S2U, geS2U. RP HPLC analysis method: 0-5 min 0% B, 5-21 min 0-80% B, 21-26 min 80-0% B

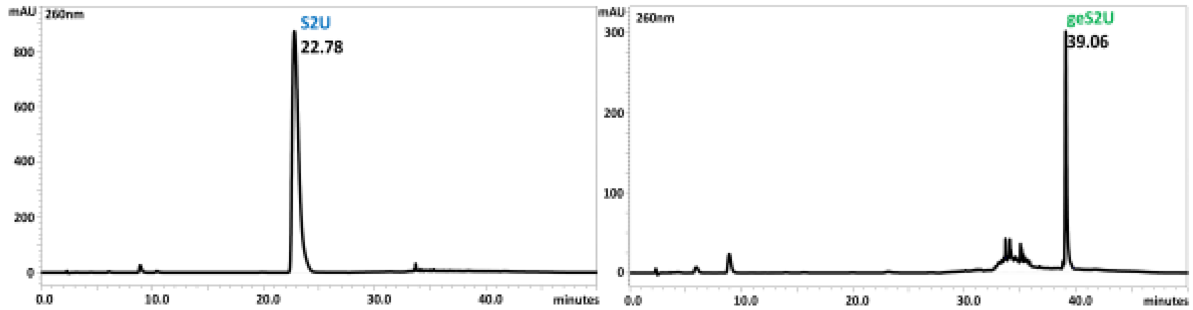

B. 3-mer RNA. RP HPLC analysis method: 0-5 min 0% B, 5-21 min 0-80% B, 21-26 min 80-0% B

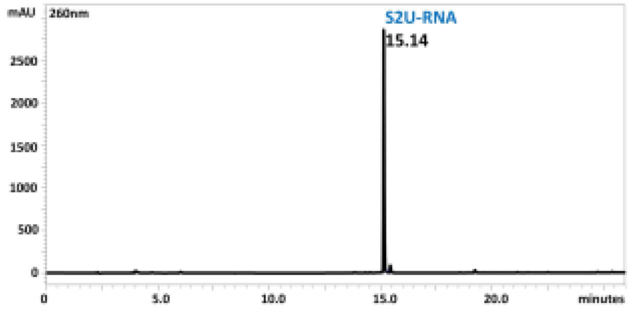

C. 7-mer RNA. RP HPLC analysis method: 0-5 min 0% B, 5-21 min 0-80% B, 21-26 min 80-0% B

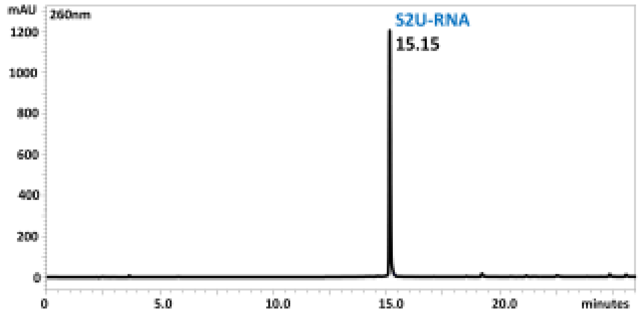

D. 17-mers RNA: S2U(33)-RNA^{Lys}; S2U(34)-RNA^{Lys}; geS2U(34)-RNA^{Lys}; mnm5S2U-RNA^{Lys}, S2U(35)-RNA^{Lys}; S2U(36)-RNA^{Lys}; S2U(34)-RNA^{Arg} RP HPLC analysis method: 0-5 min 0% B, 5-21 min 0-80% B, 21-26 min 80-0% B

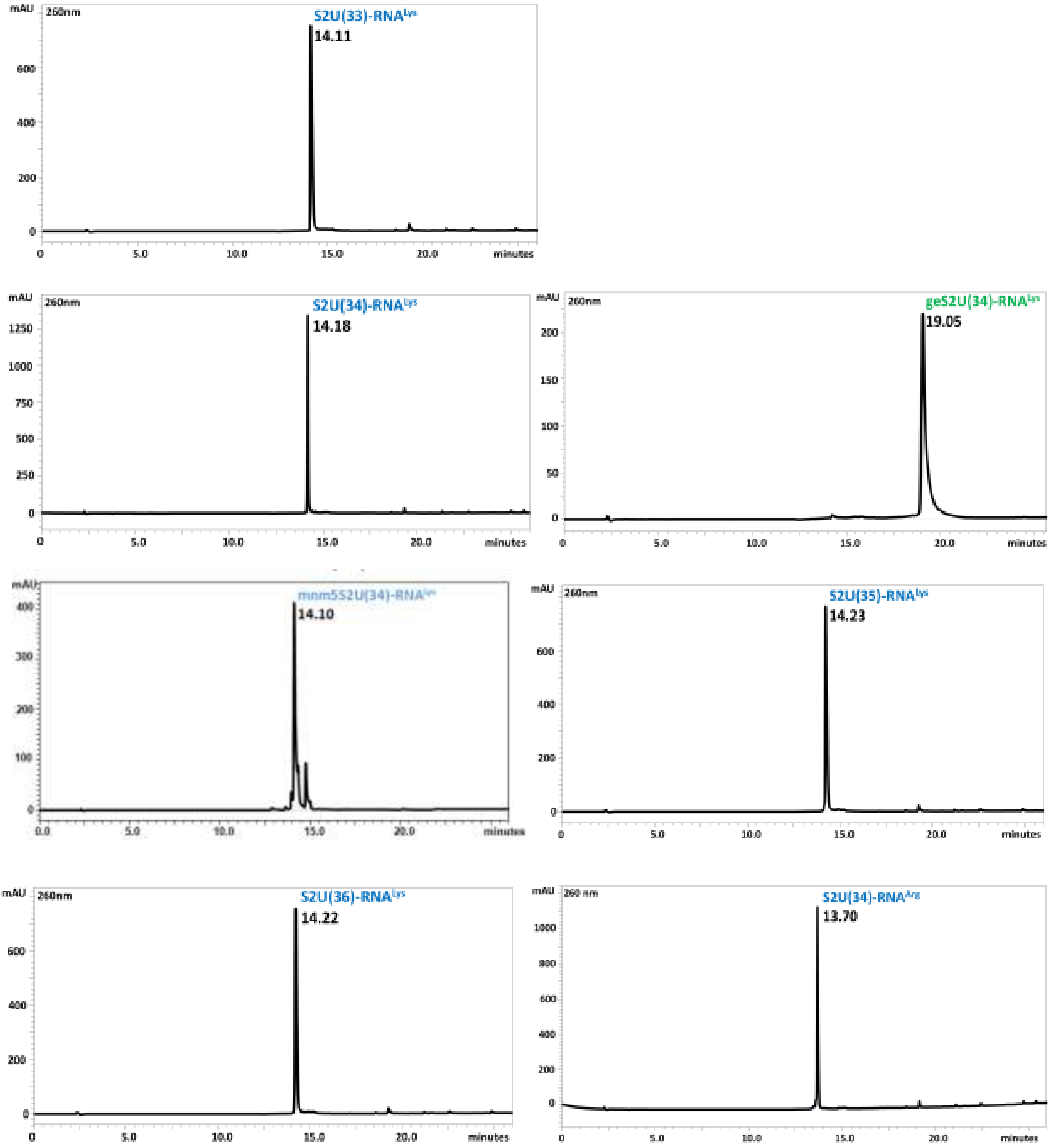

Figure S6 Geranylation of S2U-RNA substrates with the MBP-SelU enzyme (conditions described in the Table S2). Analysis by RP HPLC system (Shimadzu, Japan), Kinetex 5µ C-18 100A; 250x4.60 mm column (Phenomenex), buffers (A: 0.1 M CH_3COONH_4; B: 0.1 M CH_3COONH_4/ 40 % CH_3CN). Reaction products were separated in the linear gradient of acetonitrile: 0-5 min 0% B, 5-21 min 0-80% B, 21-26 min 80-0% B

A. Geranylation of S2U(33)-RNA, reaction yield 9.1 %

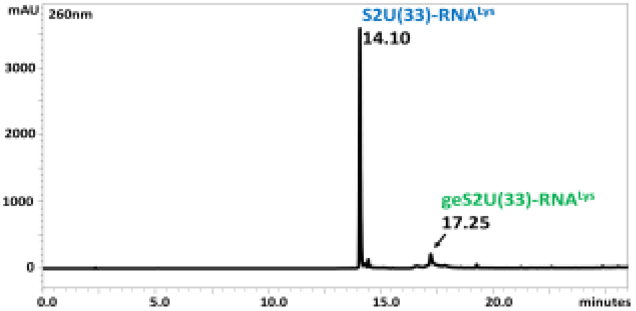

B. Geranylation of S2U(34)-RNA^{Lys}, reaction yield 91.2 %

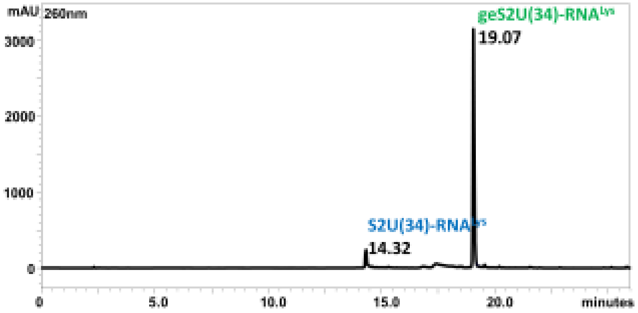

C. Geranylation of S2U(35)-RNA, reaction yield 65.4 %

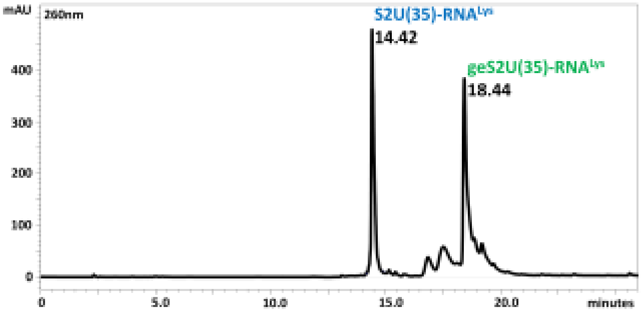

D. Geranylation of S2U(36)-RNA, reaction yield 12.8 %

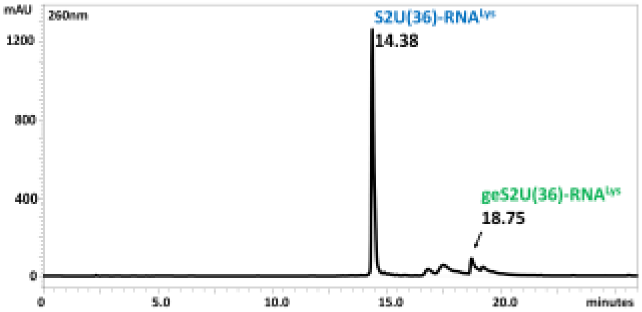

E. Geranylation of mnm5S2U-RNA^{Lys}, reaction yield 90%

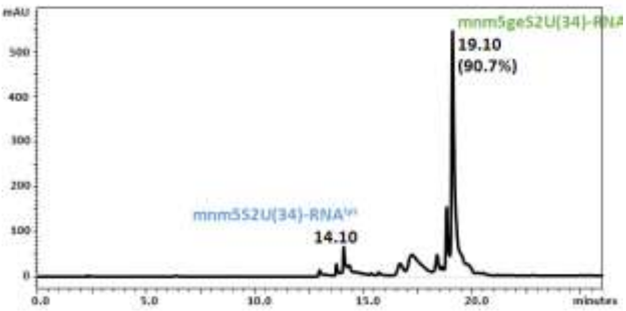

F. Geranylation of S2U(34)-RNA^{Arg}, reaction yield 13.5 %

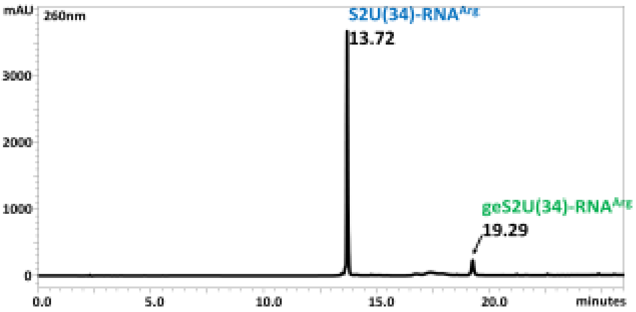

G. Geranylation of 7-mer S2U-RNA, reaction yield 24,95 %

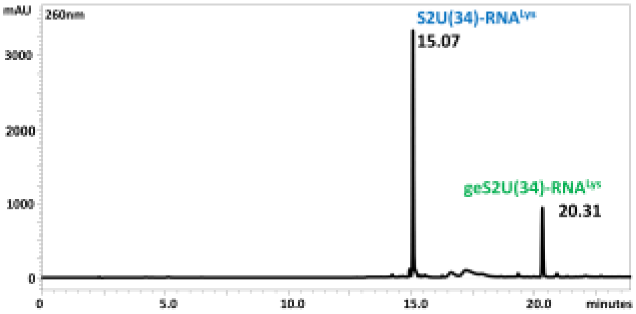

H. Geranylation of 3-mer S2U-RNA, reaction yield 14.5 %

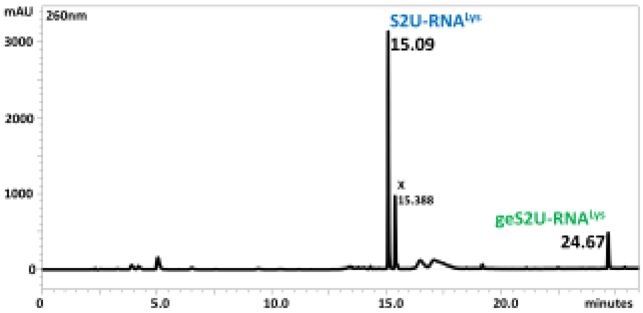

I. Geranylation of nucleoside S2U, reaction yield 0%

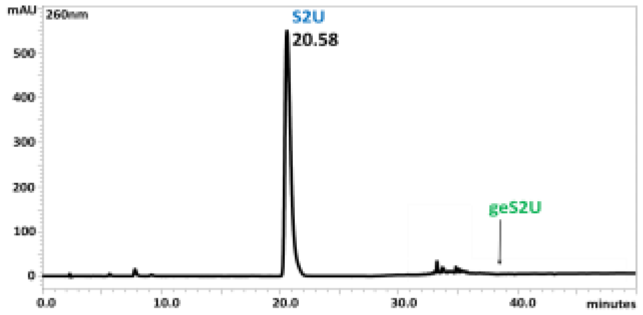

Figure S7 Selenation of geS2U-RNA substrates with the MBP-SelU enzyme (conditions described in the Table S2). Analysis by RP HPLC system (Shimadzu, Japan) using Kinetex 5µ C-18 100A; 250 x 4.60 mm column (Phenomenex) and buffers (A: 0.1 M CH_3COONH_4; B: 0.1 M CH_3COONH_4/ 40 % CH_3CN). Reaction products were separated in the linear gradient of acetonitrile: 0-5 min 0% B, 5-21 min 0-80% B, 21-26 min 80-0% B

A. Selenation of geS2U(34)-RNA^{Lys}, reaction yield 100 %

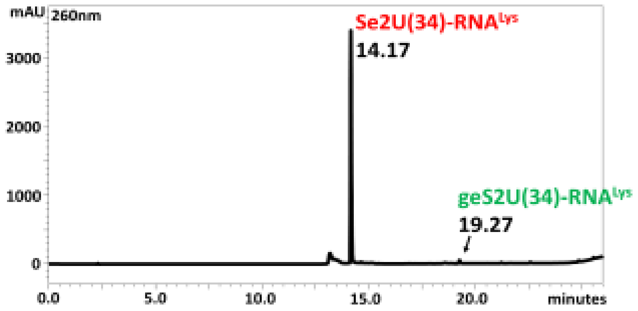

B. Selenation of mnm5geS2U(34)-RNA^{Lys}, reaction yield 100 %

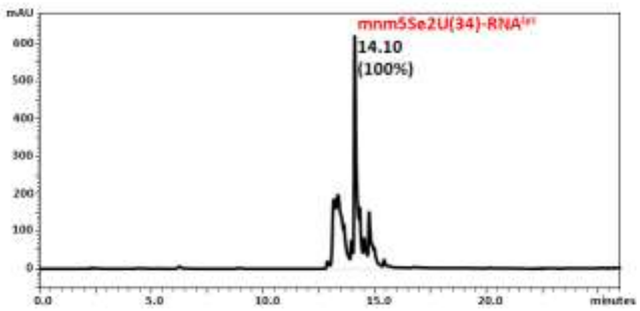

C. Selenation of geS2U nucleoside

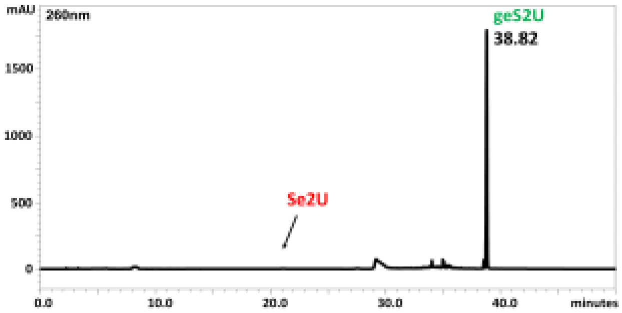

Figure S8 MST Capillary Scan test, the fluorescence intensity changes in the measured sample

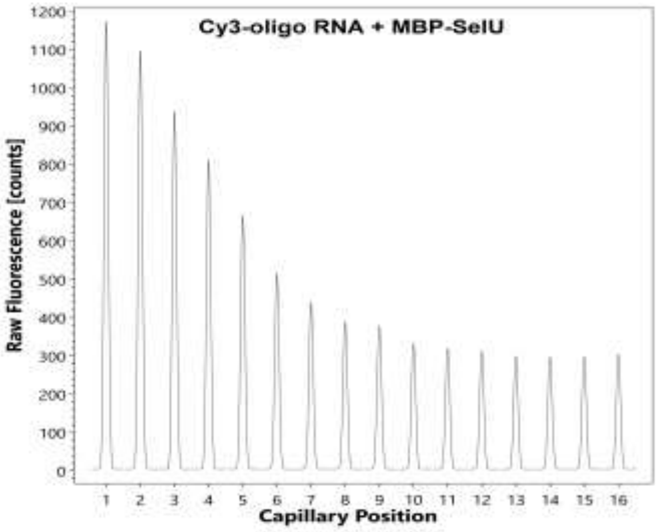

Table S4 MST Dataset Overview

**Analysis Settings**				
**Measured Settings**				
**MST-Power:**	40 %	40 %	40 %	40 %
**Excitation-Power:**	20 %	20 %	20 %	20 %
**Excitation type:**	Green	Green	Green	Green
**Thermostat Setpoint:**	22 °C	22 °C	22 °C	22 °C
**Fit Results (Kd)**				
**Fit Model:**	Kd	K_d	K_d	K_d
**Bound**	1292.4	1171.1	961.29	1482
**Unbound**	312.27	293.71	352.05	326.37
**K_d**	2.555E-05	2.2453E-05	3.9478E-06	2.7148E-05
**TargetConc**	0,000000065	0,000000065	0,00000005	0,000000075
**Standard**	17,69909363	16,5025737	20,29278566	16,21044909
**Response Amplitude**	980.15997	877.43272	609.24595	1155.6109
**Std. Error of Regression**	17.699094	16.502574	20.292786	16.210449
**Signal to Noise**	59.486702	57.113148	32.249645	76.575623
Type of Analysis	Initial fluorescence			

Figure S9 Determination of tRNA bound to MBP-SelU. (A) 15% SDS-PAGE gel of MBP-SelU after electrophoresis, stained with ethidium bromide to visualize tRNA, line 1 - protein standard, line 2 - MBP-SelU; (B) 2% agarose stained with ethidium bromide to identify the complex of tRNA associated with MBP-SelU protein, line 1 - DNA ladder, line 2 - RNA length standard (100-nt), line 3 - native MBP-SelU, line 4 - thermally denatured MBP-SelU; (C) The agarose gel from panel (B) stained with the dye Coomassie Brilliant Blue to identify the protein bands

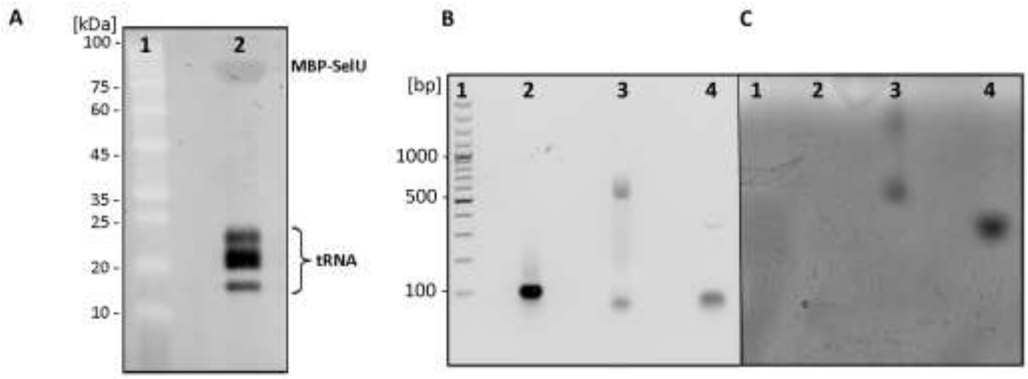

Figure S10 Sequences and the positions of the nucleoside modifications in the bacterial tRNAs specific for Lys, Glu and Gln, the underlined sequence corresponds to the anticodon sequence [38]

- **tRNA^{Lys} (tdbR00000181); 76 nt**

5'-GGGUCGUUAGCUCAGDDGGDAGAGCAGUUGACU**SUU**6APCAAUUG7XCGCAGGTPCGAAUCCUGCACGACCCACCA-3'

- **tRNA^{Glu1} (tdbR00000045); 76 nt**

5'-GUCCCCUUCGUCPAGAGGCCCAGGACACCGCCCU**SUC**/CGGCGGUAACAGGGGTPCGAAUCCCCUGGGGGACGCCA-3'

- **tRNA^{Glu2} (tdbR00000046); 76 nt**

5'-GUCCCCUUCGUCPAGAGGCCCAGGACACCGCCCU**SUC**/CGGCGGUAACAGGGGTPCGAAUCCCCUAGGGGACGCCA-3'

- **tRNA^{Glu3} (tdbR00000047); 75 nt**

5'-GUCCCCUUCGUCPAGAGGCCAGGACACCGCCCU**SUC**/CGGCGGUAACAGGGGTPCGAAUCCCCUAGGGGACGCCA-3'

- **tRNA^{Gln} (tdbR00000333); 75 nt**

5'-UGGGGUA4CGCCAAGC#GDAAGGCACCGGUJU**NUG**/PACCGGCAUUCCCUGGTPCGAAUCCAGGUACCCCAGCCA-3'

D - Dihydrouridine
S - 5-methylaminomethyl-2-geranyl-thiouridine -
6 - N6-threonylcarbamoyladenosine
P - Pseudouridine
7 - 7-methylguanosine
X - 3-(3-amino-3-carboxypropyl)uridine
T - ribosylthymine
/- 2-methyladenosine
4 - 4-thiouridine
# - 2'-O-methylguanosine
J - 2'-O-methyladenosine
N- 5-carboxymethylaminomethyl-2-geranyl thiouridine

Table S5 Calculation of the molecular formula and molecular weight of the bacterial tRNA^{Lys}, tRNA^{Glu} and tRNA^{Gln} depending on nucleoside modification at the wobble position

**tRNA^{Lys}**			
No	Name	Molecular formula	Molecular weight
1	mnm5geS2U	$C_{744}H_{939}N_{285}O_{541}P_{76}S$	24916.1
2	cmnm5geS2U	$C_{745}H_{939}N_{285}O_{543}P_{76}S$	24960.1
3	nm5geS2U	$C_{743}H_{937}N_{285}O_{541}P_{76}S$	24902.1
4	geS2U	$C_{742}H_{934}N_{284}O_{541}P_{76}S$	24873.0
5	mnm5S2U	$C_{734}H_{923}N_{285}O_{541}P_{76}S$	24779.9
6	cmnm5S2U	$C_{735}H_{923}N_{285}O_{543}P_{76}S$	24823.9
7	nm5S2U	$C_{733}H_{921}N_{285}O_{541}P_{76}S$	24765.8
8	S2U	$C_{732}H_{918}N_{284}O_{541}P_{76}S$	24736.8
9	mnm5Se2U	$C_{734}H_{923}N_{285}O_{541}P_{76}Se$	24826.8
10	cmnm5Se2U	$C_{735}H_{923}N_{285}O_{543}P_{76}Se$	24870.8
11	nm5Se2U	$C_{733}H_{921}N_{285}O_{541}P_{76}Se$	24812.7
12	Se2U	$C_{732}H_{918}N_{284}O_{541}P_{76}Se$	24783.7
13	mnm5U	$C_{734}H_{923}N_{285}O_{542}P_{76}$	24763.8
14	cmnm5U	$C_{735}H_{923}N_{285}O_{544}P_{76}$	24807.8
15	nm5U	$C_{733}H_{921}N_{285}O_{542}P_{76}$	24749.8
16	U	$C_{732}H_{918}N_{284}O_{542}P_{76}$	24720.7

**tRNA^{Glu1}**			
No	Name	Molecular formula	Molecular weight
1	mnm5geS2U	$C_{734}H_{926}N_{288}O_{532}P_{76}S$	24680.9
2	cmnm5geS2U	$C_{735}H_{926}N_{288}O_{534}P_{76}S$	24724.9
3	nm5geS2U	$C_{733}H_{924}N_{288}O_{532}P_{76}S$	24666.9
4	geS2U	$C_{732}H_{921}N_{287}O_{532}P_{76}S$	24637.8
5	mnm5S2U	$C_{724}H_{910}N_{288}O_{532}P_{76}S$	24544.7
6	cmnm5S2U	$C_{725}H_{910}N_{288}O_{534}P_{76}S$	24588.7
7	nm5S2U	$C_{723}H_{908}N_{288}O_{532}P_{76}S$	24530.7
8	S2U	$C_{722}H_{905}N_{287}O_{532}P_{76}S$	24501.6
9	mnm5Se2U	$C_{724}H_{910}N_{288}O_{532}P_{76}Se$	24591.6
10	cmnm5Se2U	$C_{725}H_{910}N_{288}O_{534}P_{76}Se$	24635.6
11	nm5Se2U	$C_{723}H_{908}N_{288}O_{532}P_{76}Se$	24577.5
12	Se2U	$C_{722}H_{905}N_{287}O_{532}P_{76}Se$	24548.5
13	mnm5U	$C_{724}H_{910}N_{288}O_{533}P_{76}$	24528.6
14	cmnm5U	$C_{725}H_{910}N_{288}O_{535}P_{76}$	24572.6
15	nm5U	$C_{723}H_{908}N_{288}O_{533}P_{76}$	24514.6
16	U	$C_{722}H_{905}N_{287}O_{533}P_{76}$	24485.5

**tRNA^{Glu2}**			
No	Name	Molecular formula	Molecular weight
1	mnm5geS2U	$C_{734}H_{926}N_{288}O_{531}P_{76}S$	24664.9
2	cmnm5geS2U	$C_{735}H_{926}N_{288}O_{533}P_{76}S$	24708.9
3	nm5geS2U	$C_{733}H_{924}N_{288}O_{531}P_{76}S$	24650.9
4	geS2U	$C_{732}H_{921}N_{287}O_{531}P_{76}S$	24621.85
5	mnm5S2U	$C_{724}H_{910}N_{288}O_{531}P_{76}S$	24528.7
6	cmnm5S2U	$C_{725}H_{910}N_{288}O_{533}P_{76}S$	24572.7
7	nm5S2U	$C_{723}H_{908}N_{288}O_{531}P_{76}S$	24514.7
8	S2U	$C_{722}H_{905}N_{287}O_{531}P_{76}S$	24485.6
9	mnm5Se2U	$C_{724}H_{910}N_{288}O_{531}P_{76}Se$	24575.6
10	cmnm5Se2U	$C_{725}H_{910}N_{288}O_{533}P_{76}Se$	24619.6
11	nm5Se2U	$C_{723}H_{908}N_{288}O_{531}P_{76}Se$	24561.5
12	Se2U	$C_{722}H_{905}N_{287}O_{531}P_{76}Se$	24532.5
13	mnm5U	$C_{724}H_{910}N_{288}O_{532}P_{76}$	24512.6
14	cmnm5U	$C_{725}H_{910}N_{288}O_{534}P_{76}$	24556.6
15	nm5U	$C_{723}H_{908}N_{288}O_{532}P_{76}$	24498.6
16	U	$C_{722}H_{905}N_{287}O_{532}P_{76}$	24469.5

**tRNA^{Glu3}**			
No	Name	Molecular formula	Molecular weight
1	mnm5geS2U	$C_{725}H_{914}N_{285}O_{524}P_{75}S$	24359.7
2	cmnm5geS2U	$C_{726}H_{914}N_{285}O_{526}P_{75}S$	24403.7
3	nm5geS2U	$C_{724}H_{912}N_{285}O_{524}P_{75}S$	24345.7
4	geS2U	$C_{723}H_{909}N_{284}O_{524}P_{75}S$	24316.7
5	mnm5S2U	$C_{715}H_{898}N_{285}O_{524}P_{75}S$	24223.5
6	cmnm5S2U	$C_{716}H_{898}N_{285}O_{526}P_{75}S$	24267.5
7	nm5S2U	$C_{714}H_{896}N_{285}O_{524}P_{75}S$	24209.5
8	S2U	$C_{713}H_{893}N_{284}O_{524}P_{75}S$	24180.4
9	mnm5Se2U	$C_{715}H_{898}N_{285}O_{524}P_{75}Se$	24270.4
10	cmnm5Se2U	$C_{716}H_{898}N_{285}O_{526}P_{75}Se$	24314.4
11	nm5Se2U	$C_{714}H_{896}N_{285}O_{524}P_{75}Se$	24256.4
12	Se2U	$C_{713}H_{893}N_{284}O_{524}P_{75}Se$	24227.3
13	mnm5U	$C_{715}H_{898}N_{285}O_{525}P_{75}$	24207.4
14	cmnm5U	$C_{716}H_{898}N_{285}O_{527}P_{75}$	24251.4
15	nm5U	$C_{714}H_{896}N_{285}O_{525}P_{75}$	24193.4
16	U	$C_{713}H_{893}N_{284}O_{525}P_{75}$	24164.4

**tRNA^{Gln}(tdbR00000333)**			
No	Name	Molecular formula	Molecular weight
1	mnm5geS2U	$C_{728}H_{917}N_{284}O_{524}P_{75}S_2$	24416.8
2	cmnm5geS2U	$C_{729}H_{917}N_{284}O_{526}P_{75}S_2$	24460.9
3	nm5geS2U	$C_{727}H_{915}N_{284}O_{524}P_{75}S_2$	24402.8
4	geS2U	$C_{726}H_{912}N_{283}O_{524}P_{75}S_2$	24373.8
5	mnm5S2U	$C_{718}H_{901}N_{284}O_{524}P_{75}S_2$	24280.6
6	cmnm5S2U	$C_{719}H_{901}N_{284}O_{526}P_{75}S_2$	24324.6
7	nm5S2U	$C_{717}H_{899}N_{284}O_{524}P_{75}S_2$	24266.6
8	S2U	$C_{716}H_{896}N_{283}O_{524}P_{75}S_2$	24237.5
9	mnm5Se2U	$C_{718}H_{901}N_{284}O_{524}P_{75}SeS$	24327.5
10	cmnm5Se2U	$C_{719}H_{901}N_{284}O_{526}P_{75}SeS$	24371.5
11	nm5Se2U	$C_{717}H_{899}N_{284}O_{524}P_{75}SeS$	24313.5
12	Se2U	$C_{716}H_{896}N_{283}O_{524}P_{75}SeS$	24284.4
13	mnm5U	$C_{718}H_{901}N_{284}O_{525}P_{75}S$	24264.5
14	cmnm5U	$C_{719}H_{901}N_{284}O_{527}P_{75}S$	24308.6
15	nm5U	$C_{717}H_{899}N_{284}O_{525}P_{75}S$	24250.5
16	U	$C_{716}H_{896}N_{283}O_{525}P_{75}S$	24221.5

Figure S11 The list of nucleoside standards used in studies, their molecular formula and UV characteristics

**Nucleoside standards**	**UV characteristic, λ_{max} [nm]**
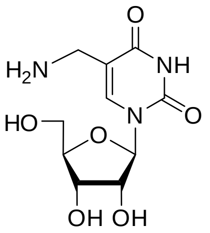<br>nm5U (MW 273.0883)	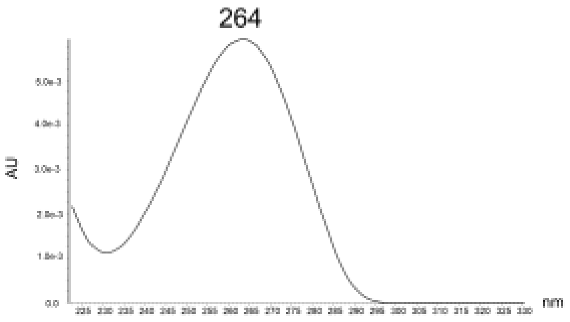
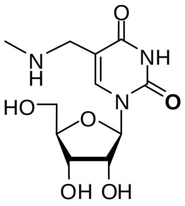<br>mnm5U (MW 287.1039)	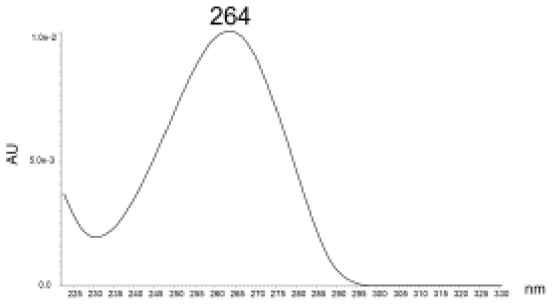
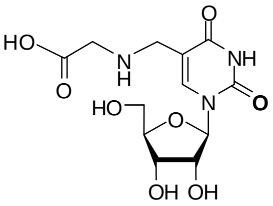<br>cmnm5U (MW 331.0938)	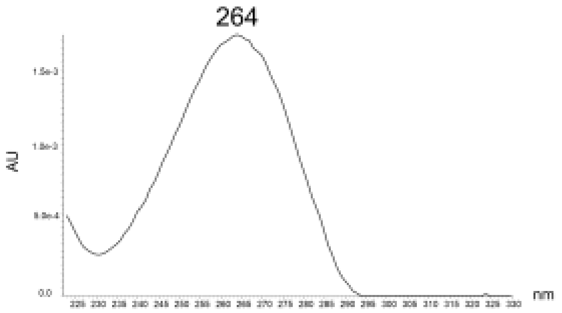
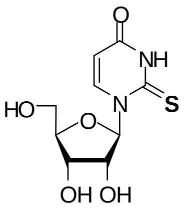<br>S2U (MW 260.0389)	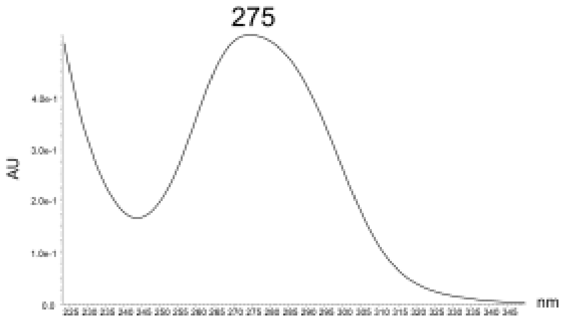

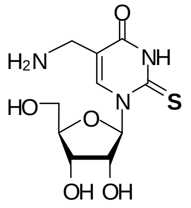<br>nm5S2U (MW 289.0654)	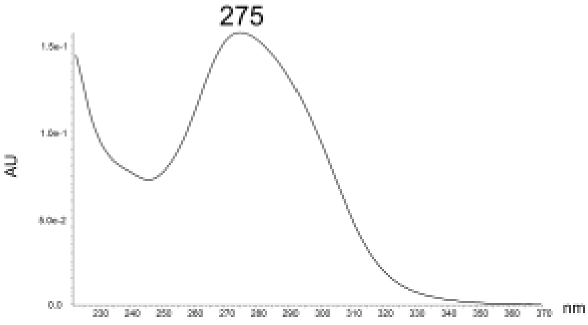
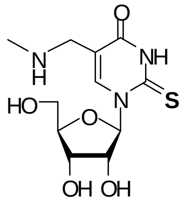<br>mnm5S2U (MW 303.0811)	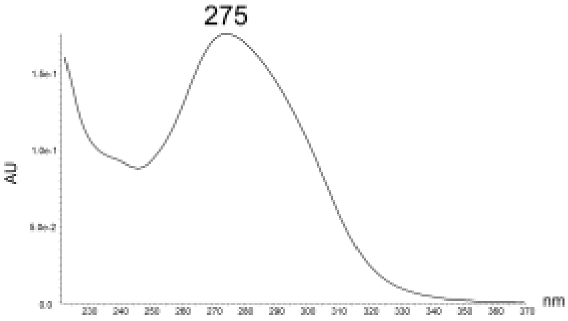
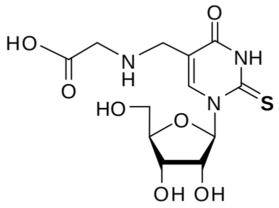<br>cmnm5S2U (MW 347.0709)	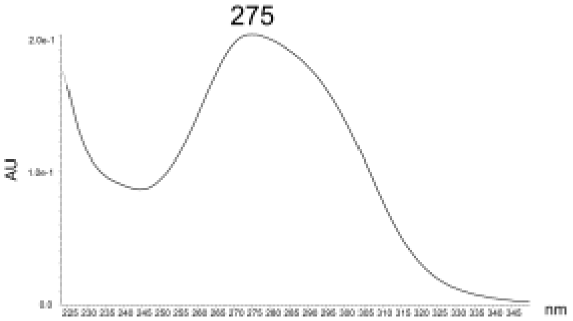
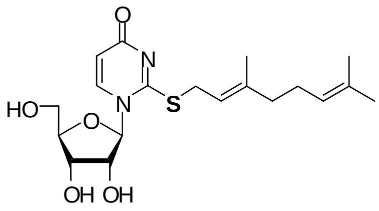<br>geS2U (MW 396.1640)	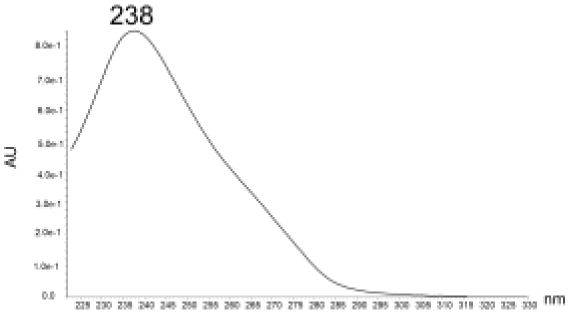
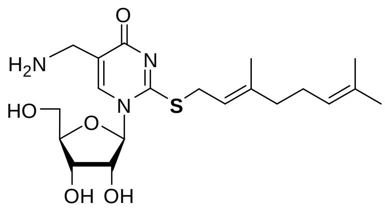<br>nm5geS2U (MW 425.1906)	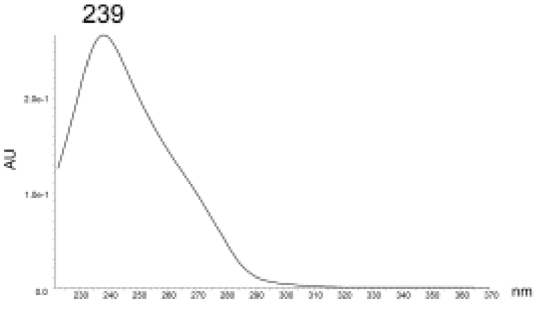

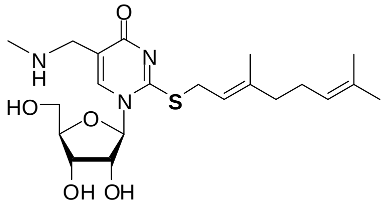<br>mnm5geS2U (MW 439.2062)	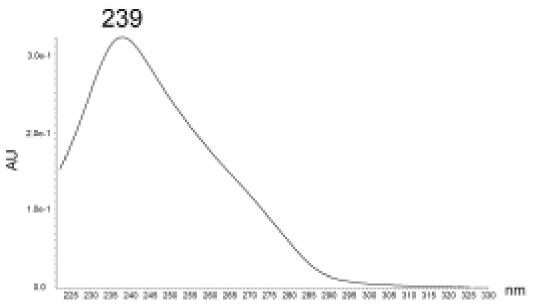
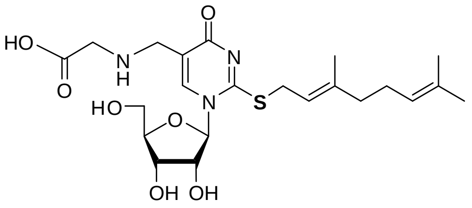<br>cmnm5geS2U (MW 483.1961)	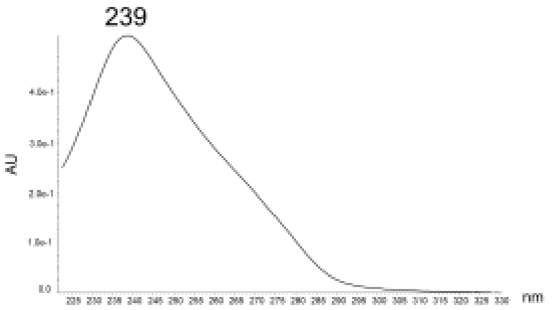
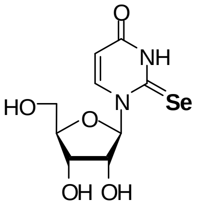<br>Se2U (MW 305.9919)	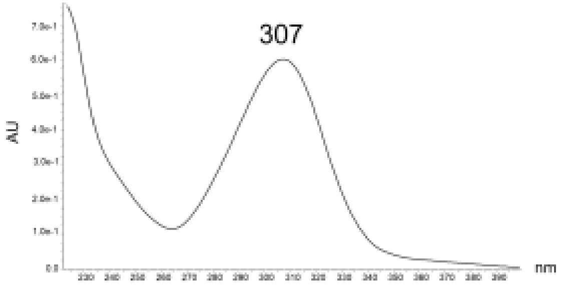
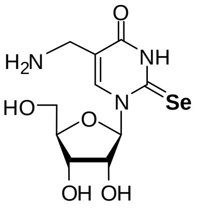<br>nm5Se2U (MW 337.0176)	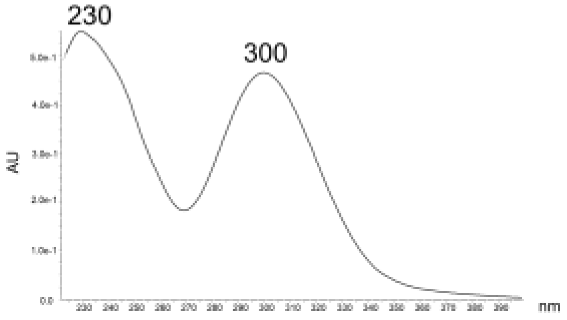
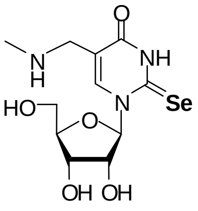<br>mnm5Se2U (MW 349.0341)	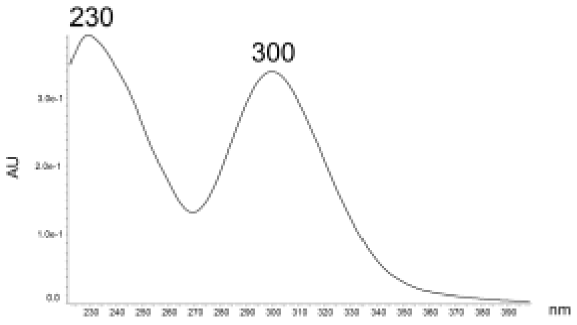

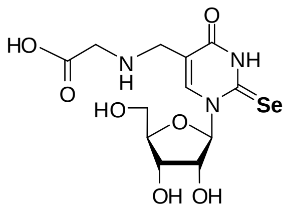

cmnm5Se2U (MW 393.0239)

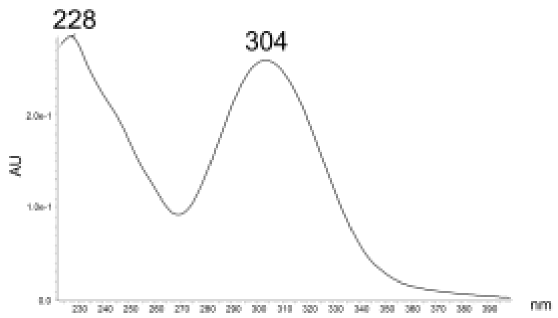

Table S6 Qualitative analysis of modified nucleosides present in the wobble position of tRNAs associated with MBP-SelU protein, results of UPLC-PDA-ESI(-)-HRMS measurements

Name of nucleoside modification	Formula	Rt [min]	*m/z* [M-H]$^-$	
			Calculated	Measured
mnm5U	$C_{11}O_6N_3H_{17}$	2.94	286.1039	286.1043
cmnm5U	$C_{12}O_8N_3H_{17}$	4.30	330.0938	330.0944
nm5U	$C_{10}O_6N_3H_{15}$	2.21	272.0883	272.0885
geS2U	$C_{19}H_{28}N_2O_5S$	18.52	395.1641	395.1640
mnm5geS2U	$C_{21}H_{33}N_3O_5S$	17.61	438.2062	438.2061
cmnm5geS2U	$C_{22}H_{33}N_3O_7S$	17.92	482.1961	482.1967
nm5geS2U	$C_{20}H_{31}N_3O_5S$	17.58	424.1906	424.1903

Table S7. Results of UPLC-PDA-ESI(-)-HRMS measurements, identification of additional (present in a position other than the wobble) modified nucleosides in the bacterial tRNAs specific for Lys, Glu and Gln associated with the MBP-SelU protein

Name of nucleoside modification	Formula	Rt [min]	*m/z* [M-H]$^-$	
			Calculated	Measured
S4U (4-thiouridine)	$C_9O_5N_2H_{12}S_1$	11.32	259.0389	259.0389
m2A (2-methyladenosine)	$C_{11}O_4N_5H_{15}$	10.93	280.1046	280.1049
Gm (2'-O-methylguanosine)	$C_{11}O_5N_5H_{15}$	11.80	296.0995	296.0992
Um (2'-O-methyluridine)	$C_{10}O_6N_2H_{14}$	9.88	257.0774	257.0779
Ψ (Pseudouridine)	$C_9O_6N_2H_{12}$	3.40	243.0632	243.0622
m5U (5-methyluridine)	$C_{10}O_6N_2H_{14}$	9.88	257.0774	257.0778
m7G (7-methylguanosine)	$C_{11}O_5N_5H_{15}$	7.85	296.0095	296.1001
t6A (N6-threonylcarbamoyladenosine)	$C_{15}O_8N_6H_{20}$	16.12	411.1264	411.1264
acp3U (3-(3-amino-3-carboxypropyl)uridine)	$C_{13}O_8N_3H_{19}$	6.44	344.1092	344.1095

Figure S12 Elemental Composition Report for mnm5S2U, mnm5geS2U and mnm5Se2U obtained in the geranylation and selenation reactions of tRNA catalyzed by MBP-SelU synthase (R5S2U-tRNA^{Lys} subjected to geranylation and selenation reactions).

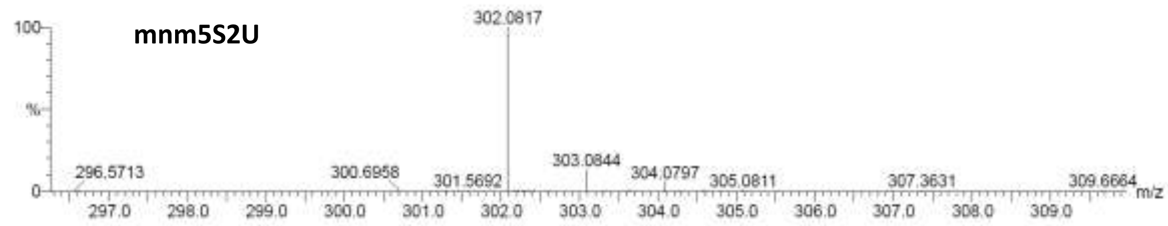

Minimum:				-1.5				
Maximum:		5.0	5.0	50.0				
Mass	Calc. Mass	mDa	PPM	DBE	i-FIT	Norm	Conf(%)	Formula
302.0817	302.0811	0.6	2.0	5.5	228.7	n/a	n/a	C11 H16 N3 O5 S

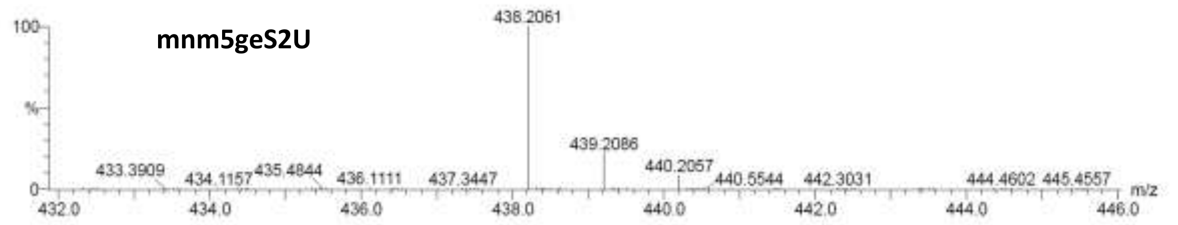

Minimum:				-1.5				
Maximum:		5.0	5.0	50.0				
Mass	Calc. Mass	mDa	PPM	DBE	i-FIT	Norm	Conf(%)	Formula
438.2061	438.2063	-0.2	-0.5	7.5	488.5	n/a	n/a	C21 H32 N3 O5 S

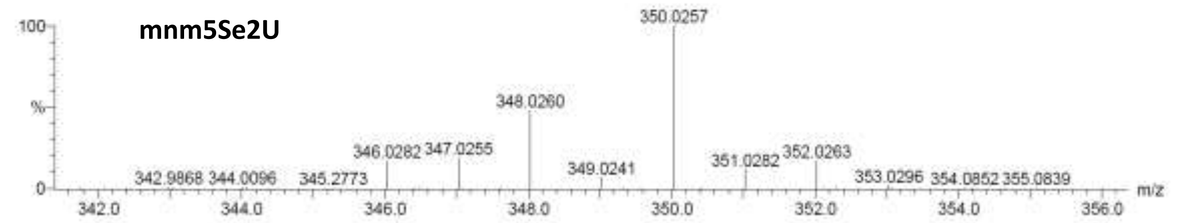

Minimum:				-1.5				
Maximum:		5.0	5.0	50.0				
Mass	Calc. Mass	mDa	PPM	DBE	i-FIT	Norm	Conf(%)	Formula
350.0257	350.0255	0.2	0.6	6.5	125.2	n/a	n/a	C11 H16 N3 O5 Se
